# Current Awareness on Comparative and Functional Genomics

**DOI:** 10.1002/cfg.60

**Published:** 2001-12


**Comparative and Functional Genomics**
*Comp. Funct. Genom.* 2001; **2:** 401–412
**DOI**:10.1002/cfg.60

### Current awareness on comparative and functional genomics

### 1 Reviews & symposia

2001. Special issue: From Genome to Proteome. Proceedings of the Vth Siena Two-Dimensional Electrophoresis Meeting - Siena, Italy - September 4-7, 2000. *Electrophoresis* **22:** (9)

2001. Special issue on genomic approaches to interactions of plants with pathogens and symbionts. *Curr Opin Plant Biol* **4:** (4)

Altman RB, Raychaudhuri S. 2001. Stanford Med Information, 251 Campus Dr, Stanford, Ca 95305, USA. Whole-genome expression analysis: challenges beyond clustering. *Curr Opin Struct Biol* **11:** (3) 340.

Anderson NG, Matheson A, Anderson NL*. 2001. *Large Scale Proteomics Corp, 9620 Med Ctr Dr, Rockville, Md 20850, USA. Back to the future: The human protein index (HPI) and the agenda for post-proteomic biology. *Proteomics* **1:** (1) 3.

Anisimov SV, Lakatta EG, Boheler KR*. 2001. *NIH/NIA, Cardiovasc Sci Lab, Baltimore, Md 21224, USA. Discovering altered genomic expression patterns in heart: Transcriptome determination by serial analysis of gene expression (Review). *Eur J Heart Fail* **3:** (3) 271.

Arrell DK, Neverova I, Van Eyk JE*. 2001. *Queen's Univ, Dept Physiol, 429 Botterell Hall, Kingston, Ontario, Canada K7L 3N6. Cardiovascular proteomics: Evolution and potential (Review). *Circ Res* **88:** (8) 763.

Bancroft I. 2001. John Innes Ctr Plant Sci Res, Dept Brassica & Oilseeds Res, Norwich Res Pk, Norwich NR4 7UH, England. Duplicate and diverge: The evolution of plant genome microstructure (Review). *Trends Genet* **17:** (2) 89.

Battersby BJ, Lawrie GA, Trau M. 2001. Univ Queensland, Dept Chem, Ctr Nanotechnol & Biomaterials, St Lucia, Qld 4072, Australia. Optical encoding of microbeads for gene screening: Alternatives to microarrays (Review). *Drug Discov Today* **6:** (12 Suppl S1) S19.

Bertucci F, Loriod B, Tagett R, Granjeaud S, Birnbaum D, Nguyen C, Houlgatte R*. 2001. *Inst J Paoli-Calmettes, Lab Biol Tumeurs, FR-13009 Marseille, France. DNA arrays: Technological aspects and applications (French, English Abstract). *Bull Cancer* **88:** (3) 243.

Bjorkholm BM, Oh JD, Falk PG, Engstrand LG, Gordon JI*. 2001. *Washington Univ, Sch Med, Dept Mol Biol & Pharmacol, Box 8103, 660 Sth Euclid Ave, St Louis, Mo 63110, USA. Genomics and proteomics converge on *Helicobacter pylori*. *Curr Opin Microbiol* **4:** (3) 237.

Boucher Y, Nesbo CL, Doolittle WF*. 2001. *Dalhousie Univ, Dept Biochem & Mol Biol, Inst Adv Res Program Evolutionary Biol, Halifax, Nova Scotia, Canada BSH 4H7. Microbial genomes: Dealing with diversity. *Curr Opin Microbiol* **4:** (3) 285.

Bremer C, Weissleder R*. 2001. *MGH, Ctr Mol Imaging Res, Bldg 149, 13th St, Boston, Ma 02129, USA. *In vivo* imaging of gene expression: MR and optical technologies. *Acad Radiol* **8:** (1) 15.

Burgess JK. 2001. Univ Sydney, Dept Pharmacol, Resp Res Grp, Bosch Bldg, Sydney, NSW 2006, Australia. Gene expression studies using microarrays. *Clin Exp Pharmacol Physiol* **28:** (4) 321.

Califano A. 2001. First Genet Trust Inc, 9 Polito Ave, Suite 930, Lyndhurst, NJ 07071, USA. Advances in sequence analysis. *Curr Opin Struct Biol* **11:** (3) 330.

Charlesworth D, Charlesworth B, McVean GAT. 2001. Univ Edinburgh, Inst Cell Anim & Populat Biol, Ashworth Lab, West Mains Rd, Kings Bldg, Edinburgh EH9 3JT, Scotland. Genome sequences and evolutionary biology, a two-way interaction (Review). *Trends Ecol Evol* **16:** (5) 235.

Charron P, Komajda M*. 2001. *Univ Paris 06, Hop La Pitie Salpetriere, Serv Cardiol, 47-83 Blvd Hop, FR-75013 Paris, France. Are we ready for pharmacogenomics in heart failure? (Review). *Eur J Pharmacol* **417:** (1-2) 1.

Cooper CS. 2001. Inst Canc Res, Haddow Labs, Cotswold Rd, Sutton SM2 5NG, England. Applications of microarray technology in breast cancer research (Review). *Breast Cancer Res Treat* **3:** (3) 158.

Cunningham MJ. 2000. Genometrix Inc, 2700 Res Forest Dr, The Woodlands, Tx 77381, USA. Genomics and proteomics: The new millenium of drug discovery and development. *J Pharmacol Toxicol Methods* **44:** (1) 291.

Davidson D, Baldock R. 2001. Western Gen Hosp, MRC Human Genet Unit, Crewe Rd, Edinburgh EH4 2XU, Scotland. Bioinformatics beyond sequence: Mapping gene function in the embryo (Review). *Nat Rev Genet* **2:** (6) 409.

Delseny M, Salses J, Cooke R, Sallaud C, Regad F, Lagoda P, Guiderdoni E, Ventelon M, Brugidou C, Ghesquiere A. 2001. Univ Perpignan, CNRS UMR 5096, Lab Genome & Dev Plantes, 52 Ave Villeneuve, FR-66860 Perpignan, France. Rice genomics: Present and future. *Plant Physiol Biochem* **39:** (3-4) 323.

Devaux F, Marc P, Jacq C*. 2001. *Ecole Normale Super, Mol Genet Lab, 46 rue Ulm, FR-75005 Paris, France. Transcriptomes, transcription activators and microarrays (Minireview). *FEBS Lett* **498:** (2-3) 140.

Deyholos MK, Galbraith DW*. 2001. *Univ Arizona, Dept Plant Sci, 1145 East 4th St, Tucson, Az 85721, USA. High density microarrays for gene expression analysis (Review). *Cytometry* **43:** (4) 229.

Ehrat M, Kresbach GM. 2001. Zeptosens AG, Benkenstr 254, CH-4018 Witterswil, Switzerland. DNA and protein microarrays and their contributions to proteomics and genomics. *Chimia* **55:** (1-2) 35.

Fisch I. 2001. Ecole Polytech Fed Lausanne, DC IGC, Ctr Biotechnol, Swiss Fed Inst Technol, SELEXIS SA, CBUE-LBTM, CH-1015 Lausanne, Switzerland. Peptide display in functional genomics. *Comb Chem High Throughput Scr* **4:** (2) 157.

Franken P, Requena N. 2001. Univ Marburg, Max-Planck-Inst Terr Mikrobiol, Karl von Frisch Str, DE-35043 Marburg, Germany. Analysis of gene expression in arbuscular mycorrhizas: New approaches and challenges (Review). *New Phytol* **150:** (3) 517.

Gaasterland T, Oprea M. 2001. Rockefeller Univ, Lab Computat Genomics, 1230 York Ave, New York, NY 10021, USA. Whole-genome analysis: Annotations and updates. *Curr Opin Struct Biol* **11:** (3) 377.

Godovac-Zimmerman J, Brown KL. 2001. UCL, Ctr Molec Med, 5 Univ St, London WC1E 6JJ, England. Perspectives for mass spectrometry and functional proteomics. *Mass Spectrom Rev* **20:** (1) 1.

Grabherr R, Ernst W. 2001. Agr Univ Vienna, Inst Appl Microbiol, Muthgasse 18, AU-1190 Vienna, Austria. The baculovirus expression system as a tool for generating diversity by viral surface display. *Comb Chem High Throughput Scr* **4:** (2) 185.

Grandi G. 2001. Chiron SpA, Dept Biol Mol, via Florentina 1, IT-53100 Siena, Italy. Antibacterial vaccine design using genomics and proteomics (Review). *Trends Biotechnol* **19:** (5) 181.

Grant WN, Viney ME. 2001. AgRes Ltd, Wallaceville Anim Res Ctr, Ward St, POB 40063, Upper Hutt, New Zealand. Post-genomic nematode parasitology. *Int J Parasitol* **31:** (9) 879.

Graveley BR. 2001. Univ Connecticut, Ctr Hlth, Dept Genet & Dev Biol, Farmington, Ct 06030, USA. Alternative splicing: Increasing diversity in the proteomic world (Review). *Trends Genet* **17:** (2) 100.

Gull K. 2001. University Manchester, School Biology Science, 2.205 Stopford Bldg, Manchester M13 9PT, England. The biology of kinetoplastid parasites: Insights and challenges from genomics and

In order to keep subscribers up-to-date with the latest developments in their field, this current awareness service is provided by John Wiley & Sons and contains newly-published material on comparative and functional genomics. Each bibliography is divided into 16 sections. 1 Reviews & symposia; 2 General; 3 Large-scale sequencing and mapping; 4 Evolutionary genomics; 5 Comparative genomics; 6 Pathways, gene families and regulons; 7 Pharmacogenomics; 8 Large-scale mutagenesis programmes; 9 Functional genomics; 10 Transcriptomics; 11 Proteomics; 12 Protein structural genomics; 13 Metabolomics; 14 Genomic approaches to development; 15 Technological advances; 16 Bioinformatics. Within each section, articles are listed in alphabetical order with respect to author. If, in the preceding period, no publications are located relevant to any one of these headings, that section will be omitted.

Copyright © 2001 John Wiley & Sons, Ltd.

post-genomics. *Int J Parasitol* **31:** (5-6) 443.
Hancock WS. 2001. Thermo Finnigan, Proteomics, 355 River Oaks Pkwy, San Jose, Ca 95134, USA. Contribution of ion trap mass spectrometers to proteomics. *Am Lab* **33:** (12) 18.
Hanke J. 2000. Pfizer Global Research & Development, Discovery Technol Ctr, Cambridge, Ma, USA. Genomics and new technologies as catalysts for change in the drug discovery paradigm. *J Law Med Ethics* **28:** (4) 15.
Harvey DJ. 2001. Oxford Glycobiol Inst, Dept Biochem, Sth Parks Rd, Oxford OX1 3QU, England. Identification of protein-bound carbohydrates by mass spectrometry (Review). *Proteomics* **1:** (2) 311.
Jenkins RE, Pennington SR*. 2001. *Univ Liverpool, Dept Human Anat & Cell Biol, New Med Sch, POB 147, Liverpool L69 3GE, England. Arrays for protein expression profiling: Towards a viable alternative to two-dimensional gel electrophoresis? *Proteomics* **1:** (1) 13.
Joussen AM, Huang S. 2001. Univ Cologne, Zentrum Augenheilkunde, Abt Netzhaut & Glaskorperchirurg, Josef Stelzmann Str 9, DE-50931 Cologne, Germany. Gene expression profiling using cDNA microarrays (Survey) (German, English Abstract). *Ophthalmologe* **98:** (6) 568.
Kagnoff MF, Eckmann L. 2001. Univ Calif San Diego, Lab Mucosal Immunol, 9500 Gilman Dr, La Jolla, Ca 92093, USA. Analysis of host responses to microbial infection using gene expression profiling. *Curr Opin Microbiol* **4:** (3) 246.
Kaufmann H, Bailey JE, Fussenegger M*. 2001. *ETH Zurich, Inst Biotechnol, CH-8093 Zurich, Switzerland. Use of antibodies for detection of phosphorylated proteins separated by two-dimensional gel electrophoresis (Review). *Proteomics* **1:** (2) 194.
Kerr MK, Churchill GA. 2001. Jackson Lab, 600 Main St, Box 303, Bar Harbor, Me 04609, USA. Statistical design and the analysis of gene expression microarray data (Review). *Genet Res* **77:** (2) 123.
Koehl P. 2001. Stanford Univ, Dept Struct Biol, Fairchild Bldg, Stanford, Ca 94305, USA. Protein structure similarities. *Curr Opin Struct Biol* **11:** (3) 348.
Kriventseva EV, Biswas M, Apweiler R. 2001. European Bioinformatics Inst, EMBL Outstn, Wellcome Trust Genome Campus, Hinxton CB10 1SD, England. Clustering and analysis of protein families. *Curr Opin Struct Biol* **11:** (3) 334.
Kuster B, Krogh TN, Mortz E, Harvey DJ. 2001. CellZome GmbH, Meyerhofstr 1, DE-69117 Heidelberg, Germany. Glycosylation analysis of gel-separated proteins (Review). *Proteomics* **1:** (2) 350.
Lagudah ES, Dubcovsky J, Powell W. 2001. CSIRO, GPO Box 1600, Canberra, ACT 2601, Australia. Wheat genomics. *Plant Physiol Biochem* **39:** (3-4) 335.
Laurell T, Nilsson J, Marko-Varga G. 2001. Lund Univ, Dept Elect Measurements, POB 134, SE-22100 Lund, Sweden. Proteomics-protein profiling technology: The trend towards a microfabricated toolbox concept. *Trends Anal Chem* **20:** (5) 225.
Lee KH. 2001. Cornell Univ, 120 Olin Hall, Ithaca, NY 14853, USA. Proteomics: A technology-driven and technology-limited discovery science (Review). *Trends Biotechnol* **19:** (6) 217.
Leeder JS. 2001. Childrens Mercy Hosp & Clin, Div Clin Pharmacol & Toxicol, Sect Dev Pharmacol & Expt Therapeut, Kansas City, Mo 64108, USA. Pharmacogenetics and pharmacogenomics. *Pediatr Clin North Am* **48:** (3) 765.
Lengeling A, Pfeffer K, Balling R. 2001. GSF, Res Ctr Hlth & Environm, Inst Expt Genet, Ingolstadter Landstr 1, DE-85764 Neuherberg, Germany. The battle of two genomes: Genetics of bacterial host-pathogen interactions in mice (Review). *Mamm Genome* **12:** (4) 261.
Lieschke GJ. 2001. Royal Melbourne Hosp, Ludwig Inst Canc Res, Melbourne Tumour Biol Branch, Cytokine Biol Lab, Melbourne, Vic 3050, Australia. Zebrafish: An emerging genetic model for the study of cytokines and hematopoiesis in the era of functional genomics. *Int J Hematol* **73:** (1) 23.
Lucchini S, Thompson A, Hinton JCD*. 2001. *Inst Food Res, Norwich Res Pk, Norwich NR4 7UA, England. Microarrays for microbiologists (Review). *Microbiology* **147:** (6) 1403.
Maggio ET, Ramnarayan K. 2001. Structural Bioinformatics Inc, San Diego, Ca 92127, USA. Recent developments in computational proteomics (Review). *Trends Biotechnol* **19:** (7) 266.
Martienssen R, McCombie WR. 2001. Cold Spring Harbor Lab, 1 Bungtown Rd, Cold Spring Harbor, NY 11724, USA. The first plant genome (Minireview). *Cell* **105:** (5) 571.
Martin JB. 2000. Harvard Univ, Fac Med, Cambridge, Ma 02138, USA. Genomics and degenerative diseases of the nervous system. *J Law Med Ethics* **28:** (4) 30.
McLeod HL, Evans WE. 2001. Washington Univ, Sch Med, Dept Med, Div Oncol, St Louis, Mo 63110, USA. Pharmacogenomics: Unlocking the human genome for better drug therapy. *Annu Rev Pharmacol Toxicol* **41:** 101.
Meltzer PS. 2001. NIH/NHGRI, Canc Genet Branch, Bethesda, Md 20892, USA. Spotting the target: Microarrays for disease gene discovery. *Curr Opin Genet Dev* **11:** (3) 258.
Miklos GLG, Maleszka R. 2001. GenetixXpress Proprietary Ltd, 78 Pacific Rd, Palm Beach, Sydney, NSW, Australia. Integrating molecular medicine with functional proteomics: Realities and expectations. *Proteomics* **1:** (1) 30.
Miklos GLG, Maleszka R. 2001. Address as above. Protein functions and biological contexts (Overview). *Proteomics* **1:** (2) 169.
Miller W. 2001. Penn State Univ, Dept Comp Sci & Engn, University Pk, Pa 16802, USA. Comparison of genomic DNA sequences: Solved and unsolved problems (Review). *Bioinformatics* **17:** (5) 391.
Nedelkov D, Nelson RW. 2001. Intrinsic Bioprobes Inc, 625 Sth Smith Rd, Tempe, Az 85218, USA. Biomolecular interaction analysis mass spectrometry: A comprehensive microscale proteomics approach. *Am Lab* **33:** (12) 22.
Niculescu AB, Kelsoe JR. 2001. Univ Calif San Diego, Dept Psychiat, La Jolla, Ca 92093, USA. Convergent functional genomics: Application to bipolar disorder. *Ann Med* **33:** (4) 263.
Norton RM. 2001. PPGX, 3500 Paramount Pkwy, Morrisville, NC 27560, USA. Clinical pharmacogenomics: Applications in pharmaceutical R&D (Review). *Drug Discov Today* **6:** (4) 180.
O'Donovan C, Apweiler R, Bairoch A. 2001. European Bioinformatics Inst, EMBL Outstn, Wellcome Trust Genome Campus, Hinxton CB10 1SD, England. The human proteomics initiative (HPI) (Review). *Trends Biotechnol* **19:** (5) 178.
Ondrej M. 2001. Acad Sci Czech Republ, Inst Plant Mol Biol, Branisovska 31, CZ-37005 Ceske Budejovice, Czech Republic. Transgenosis of *Arabidopsis thaliana* for understanding plant gene structure and functions (Review). *Biologia* **56:** (1) 1.
Payne DJ, Warren PV, Holmes DJ, Ji YD, Lonsdale JT. 2001. GlaxoSmithKline, Dept Bioinformatics, 1250 Sth Collegeville Rd, Collegeville, Pa 19426, USA. Bacterial fatty-acid biosynthesis: A genomics-driven target for antibacterial drug discovery (Review). *Drug Discov Today* **6:** (10) 537.
Quackenbush J. 2001. Inst Genomic Res, 712 Med Ctr Dr, Rockville, Md 20850, USA. Computational analysis of microarray data (Review). *Nat Rev Genet* **2:** (6) 418.
Raychaudhuri S, Sutphin PD, Chang JT, Altman RB. 2001. Stanford Univ, Dept Med, Stanford Med Informatics, 251 Campus Dr, Stanford, Ca 94305, USA. Basic microarray analysis: Grouping and feature reduction (Review). *Trends Biotechnol* **19:** (5) 189.
Read SJ, Parsons AA, Harrison DC, Philpott K, Kabnick K, O'Brien S, Clark S, Brawner M, Bates S, Gloger I *et al.* 2001. GlaxoSmithKline, Neurol Ctr Excellence Drug Discovery, New Frontiers Sci Pk Nth, Harlow CM19 5AW, England. Stroke genomics: Approaches to identify, validate, and understand ischemic stroke gene expression. *J Cereb Blood Flow Metab* **21:** (7) 755.
Rice MC, Czymmek K, Kmiec EB*. 2001. *Univ Delaware, Dept Sci Biol, Newark, De 19716, USA. The potential of nucleic acid repair in functional genomics (Review). *Nat Biotechnol* **19:** (4) 321.
Rohlff C. 2001. Oxford Glycoscience, 10 The Quadrant, Abingdon Sci Pk, Abingdon OX14 3YS, England. Proteomics in neuropsychiatric disorders. *Int J Neuropsychopharmacol* **4:** (1) 93.
Saito H, Nakajima T, Matsumoto K. 2001. Natl Children's Med Res Ctr, Dept Allergy & Immunol, Setagaya ku, 3-35-31 Taishido, Tokyo 154 8509, Japan. Human mast cell transcriptome project (Review). *Int Arch Allergy Immunol* **125:** (1) 1.
Schmitz G, Aslanidis C, Lackner KJ. 2001. Univ Hosp Regensburg, Inst Clin Chem & Lab Med, DE-93042 Regensburg, Germany. Pharmacogenomics: Implications for laboratory medicine. *Clin Chim Acta* **308:** (1-2) 43.
Schulz-Knappe P, Zucht HD, Heine C, Jurgens M, Hess R, Schrader M. 2001. BioVision GmbH & Co KG, Feodor Lynen Str 5, DE-30625 Hannover, Germany. Peptidomics: The comprehensive analysis of peptides in complex biological mixtures. *Comb Chem High Throughput Scr* **4:** (2) 207.
Sreekumar KR, Aravind L, Koonin EV. 2001. NIH/Natl Lib Med, Natl Ctr Biotechnol Information, Bethesda, Md 20894, USA. Computational

Copyright © 2001 John Wiley & Sons, Ltd. 

analysis of human disease-associated genes and their protein products. *Curr Opin Genet Dev* **11:** (3) 247.

Sternberg PW. 2001. Calif Inst Technol, Howard Hughes Inst, Div Biol, Pasadena, Ca 91125, USA. Working in the post-genomic *C. elegans* world (Minireview). *Cell* **105:** (2) 173.

Taniguchi N, Ekuni A, Ko JH, Miyoshi E, Ikeda Y, Ihara Y, Nishikawa A, Honke K, Takahashi M. 2001. Osaka Univ, Grad Sch Med, Dept Biochem, 2-2 Yamadaoka, Suita, Osaka 565 0871, Japan. A glycomic approach to the identification and characterization of glycoprotein function in cells transfected with glycosyltransferase genes (Review). *Proteomics* **1:** (2) 239.

Tarleton RL, Kissinger J. 2001. Univ Georgia, Ctr Trop & Emerging Global Dis, Athens, Ga 30602, USA. Parasite genomics: Current status and future prospects. *Curr Opin Immunol* **13:** (4) 395.

Teichmann SA, Murzin AG, Chothia C. 2001. UCL, Dept Biochem & Mol Biol, Gower St, London WC1E 6BT, England. Determination of protein function, evolution and interactions by structural genomics. *Curr Opin Struct Biol* **11:** (3) 354.

Thebault S, Machour N, Perrot F, Jouenne T, Lange C, Hubert M, Fontaine M, Tron F, Charlionet R*. 2001. *INSERM U519, Fac Med Pharm Rouen, FR-76183 Rouen, France. Object and methodological development of proteomics. *M S-Med Sci* **17:** (5) 609.

Ubarretxena-Belandia I, Engelman DM. 2001. Yale Univ, Dept Mol Biophys & Biochem, 266 Whitney Ave, POB 208114, New Haven, Ct 06520, USA. Helical membrane proteins: Diversity of functions in the content of simple architecture. *Curr Opin Struct Biol* **11:** (3) 370.

Van Belle D, Andre B. 2001. Free Univ Brussels, Inst Biol & Med Mol, Unite Bioinformatique, CP300, rue Pr Jeener & Brachet 10, BE-6041 Gosselies, Belgium. A genomic view of yeast membrane transporters (Review). *Curr Opin Cell Biol* **13:** (4) 389.

Vandenbunder B. 2001. Univ Lille 2, Inst Pasteur, CNRS FRE 2353, 1 rue Prof Calmette, BP 447, FR-59021 Lille, France. From DNA arrays to transcriptional regulatory networks (French, English Abstract). *Bull Cancer* **88:** (3) 253.

Van Oostrum J, Hoving S, Muller D, Schindler P, Steinmetz M, Towbin H, Voshol H, Wirth U. 2001. Novartis Pharma AG, Functional Genomics Area, CH-4002 Basel, Switzerland. Proteomics: From protein identification to biological function. *Chimia* **55:** (4) 354.

Van Wijk KJ. 2001. Cornell Univ, Dept Plant Biol, Emerson Bldg, 3rd Floor, Ithaca, NY 14853, USA. Challenged and prospects of plant proteomics. *Plant Physiol* **126:** (2) 501.

Vaucheret H, Fagard M. 2001. INRA, Lab Biol Cellulaire, FR-78026 Versailles, France. Transcriptional gene silencing in plants: Targets, inducers and regulators (Review). *Trends Genet* **17:** (1) 29.

Vitkup D, Melamud E, Moult J, Sander C*. 2001. *MIT, Ctr Genome Res, 1 Kendall Sq, Bldg 300, Cambridge, Ma 02139, USA. Completeness in structural genomics. *Nat Struct Biol* **8:** (6) 559.

Walch A, Specht K, Smida J, Aubele M, Zitelsberger H, Hofler H, Werner M. 2001. Tech Univ Munich, Inst Allgemeine Pathol & Pathol Anat, Ismaningr Str 22, DE-81675 Munich, Germany. Tissue microdissection techniques in quantitative genome and gene expression analyses (Review). *Histochem Cell Biol* **115:** (4) 269.

Walsh UF, Morrissey JP, O'Gara F. 2001. Natl Univ Ireland, Univ Coll Cork, BIOMERIT Res Ctr, Cork, Rep Ireland. *Pseudomonas* for biocontrol of phytopathogens: From functional genomics to commercial exploitation. *Curr Opin Biotechnol* **12:** (3) 289.

Walter G, Konthur Z, Lehrach H. 2001. Biorchard AS, Nedre Skogvei 14, NO-0281 Oslo, Norway. High-throughput screening of surface displayed gene products. *Comb Chem High Throughput Scr* **4:** (2) 193.

Wieczorek SJ, Tsongalis GJ*. 2001. *Hartford Hosp, Dept Pathol & Lab Med, 80 Seymour St, Hartford, Ct 06102, USA. Pharmacogenomics: Will it change the field of medicine? *Clin Chim Acta* **308:** (1-2) 1.

Zabarovsky ER. 2001. VA Engelhardt Mol Biol Inst, RU-119991 Moscow, Russia. Alternative approaches to genome mapping and sequencing. *Mol Biol-Engl Tr* **35:** (2) 183.

### 3 Large-scale sequencing and mapping

Akopyants NS, Clifton SW, Martin J, Pape D, Wylie T, Li L, Kissinger JC, Roos DS, Beverley SM*. 2001. *Washington Univ, Sch Med, Dept Mol Microbiol, St Louis, Mo 63110, USA. A survey of the *Leishmania major* Friedlin strain V1 genome by shotgun sequencing: A resource for DNA microarrays and expression profiling. *Mol Biochem Parasitol* **113:** (2) 337.

Aubourg S, Rouze P*. 2001. *State Univ Ghent, Flanders Interuniv Inst Biotechnol, VIB, Dept Plant Genet, BE-9000 Ghent, Belgium. Genome annotation. *Plant Physiol Biochem* **39:** (3-4) 181.

Bahr U, Darai G*. 2001. *Univ Heidelberg, Inst Med Virol, Neuenheimer Feld 324, DE-69120 Heidelberg, Germany. Analysis and characterization of the complete genome of *Tupala* (tree shrew) herpesvirus. *J Virol* **75:** (10) 4854.

Blazewicz J, Formanowicz P, Kasprzak M, Jaroszewski M, Markiewicz WT. 2001. Poznan Univ Technol, Inst Comp Sci, Piotrowo 3A, PL-60965 Poznan, Poland. Construction of DNA restriction maps based on a simplified experiment. *Bioinformatics* **17:** (5) 398.

Bolotin A, Wincker P, Mauger S, Jaillon O, Malarme K, Weissenbach J, Ehrlich SD, Sorokin A*. 2001. *INRA, Domaine Vilvert, FR-78352 Jouy-en-Josas, France. The complete genome sequence of the lactic acid bacterium *Lactococcus lactis* spp *lactis* IL1403. *Genome Res* **11:** (5) 731.

Casaregola S, Nguyen HV, Lapathitis G, Kotyk A, Gaillardin C. 2001. Inst Natl Agron Paris Grignon, INRA UMR 216, Collect Levures Interet Biotechnol, CNRS, URA 1925, FR-78850 Thiverval Grignon, France. Analysis of the constitution of the beer yeast genome by PCR, sequencing and subtelomeric sequence hybridization. *Int J Syst Evol Microbiol* **51:** (4) 1607.

Chambaud I, Heilig R, Ferris S, Barbe V, Samson D, Galisson F, Moszer I, Dybvig K, Wroblewski H, Viari A, Rocher EPC, Blanchard E*. 2001. *Univ Bordeaux 2, INRA, Inst Biol Vegetale Mol, 71 Ave Edouard Bourleaux, BP 81, FR-33883 Villenave d'Ornon, France. The complete genome sequence of the murine respiratory pathogen *Mycoplasma pulmonis*. *Nucleic Acids Res* **29:** (10) 2145.

Ferretti JJ, McShan WM, Ajdic D, Savic DJ, Lyon K, Primeaux C, Sezate S, Suvorov AN, Kenton S *et al.* 2001. Univ Oklahoma, Hlth Sci Ctr, Dept Microbiol & Immunol, Oklahoma City, Ok 73190, USA. Complete genome sequence of an M1 strain of *Streptococcus pyogenes*. *Proc Natl Acad Sci U S A* **98:** (8) 4658.

Frohme M, Camargo AA, Czink C, Matsukuma AY, Simpson AJG, Hoheisel JD, Verjovski-Almeida S. 2001. Deutsch Krebsforschungszentrum, Funct Genome Anal, DE-6900 Heidelberg, Germany. Directed gap closure in large-scale sequencing projects. *Genome Res* **11:** (5) 901.

Glusman G, Yanai I, Rubin I, Lancet D*. 2001. *Weizmann Inst Sci, Dept Mol Genet, IL-76100 Rehovot, Israel. The complete human olfactory subgenome. *Genome Res* **11:** (5) 685.

Gordon SV, Eiglmeier K, Garnier T, Brosch R, Parkhill J, Barrell B, Cole ST, Hewinson RG. 2001. Vet Labs Agcy, Woodham Lane, Addlestone KT15 3NB, England. Genomics of *Mycobacterium bovis*. *Tuberculosis* **81:** (1-2) 157.

Gray WL, Starnes B, White MW, Mahalingam R. 2001. Univ Arkansas Med Sci, Dept Microbiol & Immunol, 4301 West Markham St, Little Rock, Ar 72205, USA. The DNA sequence of the simian varicella virus genome. *Virology* **284:** (1) 123.

Hall D, Bhandarkar SM*, Arnold J, Jiang TZ. 2001. *Univ Georgia, Dept Comput Sci, Athens, Ga 30602, USA. Physical mapping with automatic capture of hybridization data. *Bioinformatics* **17:** (3) 205.

Harley E, Bonner A, Goodman N. 2001. Univ Toronto, Dept Comput Sci, 100 Coll St, Toronto, Ontario, Canada M5S 1A4. Uniform integration of genome mapping data using intersection graphs. *Bioinformatics* **17:** (6) 487.

Hauser NC, Fellenberg K, Gil R, Bastuck S, Hoheisel JD, Perez-Ortin JE*. 2001. *Univ Valencia, Dept Bioquim & Biol Molec, Dr Moliner 50, ES-46100 Burjassot, Spain. Whole genome analysis of a wine yeast strain. *Comp Funct Genom* **2:** (2) 69.

Janssen CS, Barrett MP, Lawson D, Quail MA, Harris D, Bowman S, Phillips RS, Turner CMR. 2001. Univ Glasgow, IBLS, Div Infect & Immun, Glasgow G12 8QQ, Scotland. Gene discovery in *Plasmodium chabaudi* by genome survey sequencing. *Mol Biochem Parasitol* **113:** (2) 251.

Karlin S, Bergman A, Gentles AJ. 2001. Stanford Univ, Dept Math, Serra St, Stanford, Ca 94305, USA. Annotation of the *Drosophila* genome. *Nature* **411:** (6835) 259.

Kerscher S, Durstewitz G, Casaregola S, Gaillardin C, Brandt U. 2001. Univ Klin Frankfurt, Inst Biochem 1, Zentrum Biol Chem, Theodor-Stern-Kai 7, Haus 25 B, DE-60590 Frankfurt am Main, Germany. The complete mitochondrial genome of *Yarrowia lipolytica*. *Comp Funct Genom* **2:** (2) 80.

Kuroda M, Ohta T, Uchiyama I, Baba T, Yuzawa H, Kobayashi I, Cui LZ, Oguchi A, Aoki K, Nagai Y *et al.* 2001. c/o Haramatsu K,

Copyright © 2001 John Wiley & Sons, Ltd. 

Juntendo Univ, Dept Bacteriol, Bunkyo ku, 2-1-1 Hongo, Tokyo 113 8421, Japan. Whole genome sequencing of meticillin-resistant *Staphylococcus aureus*. *Lancet* **357:** (9264) 1225.

Louis A, Ollivier E, Aude JC, Risler EL. 2001. Univ Versailles, Lab Genome & Informatique, FR-78035 Versailles, France. Massive sequence comparisons as a help in annotating genomic sequences. *Genome Res* **11:** (7) 1296.

Maddox JF, Davies KP, Crawford AM, Hulme DJ, Vaiman D, Cribiu EP, Freking BA, Beh KJ, Cockett NE, Kang N *et al.* 2001. Univ Melbourne, Ctr Anim Biotechnol, Parkville, Vic 3052, Australia. An enhanced linkage map of the sheep genome comprising more than 1000 loci. *Genome Res* **11:** (7) 1275.

McCarter JP, Bird DM, Clifton SW, Waterston RH. 2000. Washington Univ, Sch Med, Dept Genet, Genome Sequencing Ctr, Box 8501, St Louis, Mo 63108, USA. Nematode gene sequences, December 2000 update. *J Nematol* **32:** (4) 331.

Ming R, Moore PH, Zee F, Abbey CA, Ma H, Paterson AH. 2001. Hawaii Agr Res Ctr, Aiea, Hi 97601, USA. Construction and characterization of a papaya BAC library as a foundation for molecular dissection of a tree-fruit genome. *Theor Appl Genet* **102:** (6-7) 892.

Nouzova M, Neumann P, Navratilova A, Galbraith DW, Macas J*. 2001. *Univ Arizona, Dept Plant Sci, 303 Forbes, Tucson, Az 85721, USA. Microarray-based survey of repetitive genomic sequences in *Vicia* spp. *Plant Mol Biol* **45:** (2) 229.

Samretwanich K, Kittipakom K, Chiemsombat P, Ikegami M*. 2001. *Tokyo Univ Agr & Technol, Dept Biosci, Setagaya ku, 1-1-1 Sakuragaoka, Tokyo 156 8502, Japan. Complete nucleotide sequence and genome organization of soybean crinkle leaf virus. *J Phytopathol* **149:** (6) 333.

Seki M, Narusaka M, Yamaguchi-Shinozaki K, Carninci P, Kawai J, Hayashizaki Y, Shinozaki K*. 2001. *RIKEN, GSC, Plant Funct Genomics Res Grp, Plant Mutat Explorat Team, 3-1-1 Koyadai, Tsukuba, Ibaraki 305 0074, Japan. *Arabidopsis* encyclopedia using full-length cDNAs and its application. *Plant Physiol Biochem* **39:** (3-4) 211.

She Q, Singh RK, Confalonieri F, Zivanovic Y, Allard G, Awayez MJ, Chan-Weiher CCY, Clausen IG, Curtis BA, De Moors A *et al.* 2001. c/o Garrett RA, Univ Copenhagen, Inst Mol Biol, Microbial Genome Grp, Solvgade 83H, DK-1307 Copenhagen, Denmark. The complete genome of the crenarchaeon *Sulfolobus solfataricus* P2. *Proc Natl Acad Sci U S A* **98:** (14) 7835.

Tettelin H, Nelson KE, Paulsen IT, Eisen JA, Read TD, Peterson S, Heidelberg J, De Boy RT, Haft DH, Dodson RJ *et al.* 2001. c/o Fraser CM, Inst Genome Res TIGR, 9712 Med Ctr Dr, Rockville, Md 20850, USA. Complete genome sequence of a virulent isolate of *Streptococcus pneumoniae*. *Science* **293:** (5529) 498.

Von Nickisch-Rosenegk M, Brown WM, Boore JL. 2001. Humboldt Univ, Philippstr 13, DE-10115 Berlin, Germany. Complete sequence of the mitochondrial genome of the tapeworm *Hymenolepsis diminuta*: Gene arrangements indicate that platyhelminths are eutrochozoans. *Mol Biol Evol* **18:** (5) 721.

Zhang HB, Wu CC. 2001. Texas A&M Univ, Dept Soil & Crop Sci, College Station, Tx 77843, USA. BAC as tools for genome sequencing. *Plant Physiol Biochem* **39:** (3-4) 195.

### 4 Evolutionary genomics

Baisnee PF, Baldi P*, Brunak S, Pedersen AG. 2001. *Univ Calif Irvine, Dept Informat & Comp Sci, Irvine, Ca 92697, USA. Flexibility of the genetic code with respect to DNA structure. *Bioinformatics* **17:** (3) 237.

Handa N, Nakayama Y, Sadykov M, Kobayashi I*. 2001. *Univ Tokyo, Inst Med Sci, Dept Mol Biol, Tokyo 108 8639, Japan. Experimental genome evolution: Large-scale genome rearrangements associated with resistance to replacement of a chromosomal restriction-modification gene complex. *Mol Microbiol* **40:** (4) 932.

Jakobsen IB, Saleeba JA, Poidinger M, Littlejohn TG*. 2001. *Univ Sydney, Australian Genomic Information Ctr, Sydney, NSW 2006, Australia. TreeGeneBrowser: Phylogenetic data mining of gene sequences from public databases. *Bioinformatics* **17:** (6) 535.

Kreil DP, Ouzounis CA. 2001. Univ Cambridge, Wellcome Trust Genome Campus, Cambridge CB10 1SD, England. Identification of thermophilic species by the amino acid compositions deduced from their genomes. *Nucleic Acids Res* **29:** (7) 1608.

Kundu S, Bandyopadhyay D, Thakur AR*. 2001. *Univ Calcutta, Univ Coll Sci & Technol, Dept Biophys Mol Biol & Genet, 92 APC Rd, IN-700009 Calcutta, India. Sequence-based structural signatures of genome evolution. *Indian J Biochem Biophys* **38:** (1-2) 104.

Lecompte O, Ripp R, Puzos-Barbe V, Duprat S, Heilig R, Dietrich J, Thierry JC, Poch O*. 2001. *CU Strasbourg, UPR 9004, Inst Genet & Biol Mol & Cellulaire, Illkirch Graffenstaden, France. Genome evolution at the genus level: Comparison of three complete genomes of hyperthermophilic Archaea. *Genome Res* **11:** (6) 981.

Ledent V, Vervoort M*. 2001. *Ctr Genet Mol, CNRS UPR 2067, FR-91198 Gif-sur-Yvette, France. The basic helix-loop-helix protein family: Comparative genomics and phylogenetic analysis. *Genome Res* **11:** (5) 754.

Marais G, Mouchiroud D, Duret L*. 2001. *Univ Lyon 1, CNRS, Unite Mixte Rech 5558, Lab Biometrie & Biol Evolut, Bat 711, FR-69622 Villeurbanne, France. Does recombination improve selection on codon usage? Lessons from nematode and fly complete genomes. *Proc Natl Acad Sci U S A* **98:** (10) 5688.

McGuire G, Denham MC, Balding DJ. 2001. Univ Reading, Dept Appl Stat, POB 240, Reading RG6 6FN, England. MAC5: Bayesian inference of phylogenetic trees from DNA sequences incorporating gaps. *Bioinformatics* **17:** (5) 479.

Nevo E. 2001. Univ Haifa, Inst Evolut, IL-31905 Haifa, Israel. Evolution of genome-phenome diversity under environmental stress. *Proc Natl Acad Sci U S A* **98:** (11) 6233.

Rocha EPC, Danchin A, Viari A. 2001. Univ Paris 05, FR-75005 Paris, France. Evolutionary role of restriction/modification systems as revealed by comparative genome analysis. *Genome Res* **11:** (6) 946.

Ronneberg TA, Freeland SJ*, Landweber LF. 2001. *Princeton Univ, Dept Ecol & Evolutionary Biol, Princeton, NJ 08544, USA. *Genview* and *Gencode*: A pair of programs to test theories of genetic code evolution. *Bioinformatics* **17:** (3) 280.

Rujan T, Martin W. 2001. Univ Dusseldorf, Inst Bot 3, Universitatsstr 1, DE-40225 Dusseldorf, Germany. How many genes in *Arabidopsis* come from cyanobacteria? An estimate from 386 protein phylogenies. *Trends Genet* **17:** (3) 113.

Salzberg SL, White O, Peterson J, Eisen JA. 2001. Inst Genome Res, 9712 Med Ctr Dr, Rockville, Md 20850, USA. Microbial genes in the human genome: Lateral transfer or gene loss? *Science* **292:** (5523) 1903.

Suyama M, Bork P. 2001. Eur Mol Biol Lab, Meyerhofstr 1, DE-69012 Heidelberg, Germany. Evolution of prokaryotic gene order: Genome rearrangements in closely related species. *Trends Genet* **17:** (1) 10.

Tolou HJG, Couissinier-Paris P, Durand JP, Mercier V, De Pina JJ, De Micco P, Billoir F, Charrel RN, De Lamballerie X. 2001. Univ Mediterranee, Inst Med Trop, Serv Sante Armees Unite Virol Trop, Unite Virus Emergents, BP 46, FR-13998 Marseille, France. Evidence for recombination in natural populations of dengue virus type 1 based on the analysis of complete genome sequences. *J Gen Virol* **82:** (6) 1283.

Tu ZC, Dewhirst FE, Blaser MJ*. 2001. *NYU, Sch Med, Dept Med, 550 1st Ave, New York, NY 10016, USA. Evidence that the *Campylobacter fetus* sap locus is an ancient genomic constituent with origins before mammals and reptiles diverged. *Infect Immun* **69:** (4) 2237.

Vicient CM, Jaaskelainen MJ, Kalendar R, Schulman AH*. 2001. *Univ Helsinki, Plant Genomic Lab, Inst Biotechnol, Viikki Bioctr, POB 56, Viikinkaari 6, FI-00014 Helsinki, Finland. Active retrotransposons are a common feature of grass genomes. *Plant Physiol* **125:** (3) 1283.

Zoldos V, Siljak-Yakovlev S, Papes D, Sarr A, Panaud O*, Zoldos V, Papes D. 2001. *Univ Paris Sud 11, CNRS, Lab Evolut & Systemat, Orsay, France. Representational difference analysis reveals genomic differences between *Q. robur* and *Q. suber*: Implications for the study of genome evolution in the genus *Quercus*. *Mol Genet Genomics* **265:** (2) 234.

### 5 Comparative genomics

Akin A, Lin TL*, Wu CC, Bryan TA, Hooper T, Schrader D. 2001. *Purdue Univ, Dept Vet Pathobiol, West Lafayette, In 47907, USA. Nucleocapsid protein gene sequence analysis reveals close genomic relationship between turkey coronavirus and avian infectious bronchitis virus. *Acta Virol* **45:** (1) 31.

Desiere F, Mahanivong C, Hillier AJ, Chandry PS, Davidson BE, Brussow H*. 2001. *Nestec Ltd, Nestle Res Ctr, Vers-Chez-les-Blanc, CH-1000 Lausanne 26, Switzerland. Comparative genomics of

Copyright © 2001 John Wiley & Sons, Ltd.

lactococcal phages: Insight from the complete genome sequence of *Lactococcus lactis* phase BK5-T. *Virology* **283:** (2) 240.
Draye X, Lin YR, Qian XY, Bowers JE, Burow GB, Morrell PL, Peterson DG, Presting GG, Ren SX, Wing RA, Paterson AH*. 2001. *Univ Georgia, Dept Soil & Crop Sci, Appl Genet Technol Ctr, Athens, Ga 30602, USA. Toward integration of comparative genetic, physical, diversity, and cytomolecular maps for grasses and grains, using the sorghum genome as a foundation. *Plant Physiol* **125:** (3) 1325.
Dubcovsky J, Ramakrishna W, San Miguel PJ, Busso CS, Yan LL, Shiloff BA, Bennetzen JL*. 2001. *Purdue Univ, Dept Biol Sci, West Lafayette, In 47907, USA. Comparative sequence analysis of colinear barley and rice bacterial artificial chromosomes. *Plant Physiol* **125:** (3) 1342.
Ito H, Sugiyama M, Masubuchi K, Mori Y, Minamoto N*. 2001. *Gifu Univ, Fac Agr, Dept Vet Publ Hlth, 1-1 Yanagido, Gifu 501 1193, Japan. Complete nucleotide sequence of a group A avian rotavirus genome and a comparison with its counterparts of mammalian rotaviruses. *Virus Res* **75:** (2) 123.
Janke B, Dobrindt U, Hacker J, Blum-Oehler G*. 2001. *Univ Wurzburg, Inst Mol Infekt Biol, Rontgenring 11, DE-97070 Wurzburg, Germany. A subtractive hybridisation analysis of genomic differences between the uropathogenic *E. coli* strain 536 and the *E. coli* K12 strain MG1655. *FEMS Microbiol Lett* **199:** (1) 61.
Mayer K, Murphy G, Tarchini R, Wambutt R, Volckaert G, Pohl T, Dusterhoft A, Stiekema W, Entian KD, Terryn N *et al.* 2001. c/o Bancroft I, John Innes Ctr Plant Sci Res, Norwich NR7 4UH, England. Conservation of microstructure between a sequenced region of the genome of rice and multiple segments of the genome of *Arabidopsis thaliana*. *Genome Res* **11:** (7) 1167.
Rossberg M, Theres K, Acarkan A, Herrero R, Schmitt T, Schumacher K, Schmitz G, Schmidt R*. 2001. *Max-Planck-Gesell, Max Delbruck Lab, DE-50829 Cologne, Germany. Comparative sequence analysis reveals extensive microcolinearity in the lateral suppressor regions of the tomato, *Arabidopsis*, and *Capsella* genomes. *Plant Cell* **13:** (4) 979.
Schmidt R, Acarkan A, Boivin K. 2001. Max Planck Gesell, Max Delbruck Lab, Carl von Linne Weg 10, DE-50829 Cologne, Germany. Comparative structural genomics in the Brassicaceae family. *Plant Physiol Biochem* **39:** (3-4) 253.
Yanai I, Derti A, De Lisi C*. 2001. *Boston Univ, Bioinformatics Grad Program, Boston, Ma 02215, USA. Genes linked by fusion events are generally of the same functional category: A systematic analysis of 30 microbial genomes. *Proc Natl Acad Sci U S A* **98:** (14) 7940.

### 6 Pathways, gene families and regulons

Heger A, Holm L. 2001. EMBL-EBI, Struct Genomics Grp, Cambridge CB10 1SD, England. Picasso: Generating a covering set of protein family profiles. *Bioinformatics* **17:** (3) 272.
Mayfield JA, Fiebig A, Johnstone SE, Preuss D*. 2001. *Univ Chicago, Dept Mol Genet & Cell Biol, 920 East 58th St, Chicago, Il 60637, USA. Gene families from the *Arabidopsis thaliana* protein pollen coat proteome. *Science* **292:** (5526) 2482.
Myung K, Chen C, Kolodner RD*. 2001. *Univ Calif San Diego, Sch Med, Ctr Canc, Ludwig Inst Canc Res, La Jolla, Ca 92093, USA. Multiple pathways co-operate in the suppression of genome instability in *Saccharomyces cerevisiae*. *Nature* **411:** (6841) 1073.
Park J, Lappe M, Teichmann SA*. 2001. *UCL, Dept Biochem & Mol Biol, Gower St, Darwin Bldg, London WC1E 6BT, England. Mapping protein family interactions: Intramolecular and intermolecular protein family interaction repertoires in the PDB and yeast. *J Mol Biol* **307:** (3) 929.
Robinson-Rechavi M, Marchand O, Escriva H, Bardet PL, Zelus D, Hughes S, Laudet V. 2001. Ecole Normale Super Lyon, Lab Biol Mol & Cellulaire, CNRS UMR 5665, 46 Allee Italie, FR-69364 Lyon 07, France. Euteleost fish genomes are characterized by expansion of gene families. *Genome Res* **11:** (5) 781.
Shoop E, Silverstein KAT, Johnson JE, Retzel EF. 2001. Univ Minnesota, Acad Hlth Ctr, Comp Biol Ctr, Mayo Mail Code 420, Delaware St SE, Minneapolis, Mn 55455, USA. MetaFam: A unified classification of protein families. II. Schema and query capabilities. *Bioinformatics* **17:** (3) 262.
Silverstein KAT, Shoop E, Johnson JE, Retzel EF. 2001. Univ Minnesota, Acad Hlth Ctr, Comp Biol Ctr, Mayo Mail Code 43, 420 Delaware St SE, Minneapolis, Mn 55455, USA. MetaFam: A unified classification of protein families. I. Overview and statistics. *Bioinformatics* **17:** (3) 249.
Suzuki Y, Tsunoda T, Sese J, Taira H, Mizushima-Sugano J, Hata H, Ota T, Isogai T, Tanaka T, Nakamura Y *et al.* 2001. Univ Tokyo, Inst Med Sci, Dept Virol, Minato ku, Tokyo 108 8639, Japan. Identification and characterization of the potential promoter regions of 1031 kinds of human genes. *Genome Res* **11:** (5) 677.

### 7 Pharmacogenomics

Akiyoshi S, Ishii M, Nemoto N, Kawabata M*, Aburatani H, Miyazono K. 2001. *JFCR, Inst Canc, Dept Biochem, Toshima ku, 1-37-1 Kami, Tokyo 170 8455, Japan. Targets of transcriptional regulation by transforming growth factor-β: Expression profile analysis using oligonucleotide arrays. *Jpn J Cancer Res* **92:** (3) 257.
Behr MA. 2001. Montreal Gen Hosp, 1650 Cedar Ave, Montreal, Quebec, Canada H3G 1A4. Correlation between BCG genomics and protective efficacy. *Scand J Infec Dis* **33:** (4) 249.
Behr MA. 2001. Address as above. Comparative genomics of BCG vaccines. *Tuberculosis* **81:** (1-2) 165.
Bertucci F, Houlgatte R, Nguyen C, Benziane A, Nasser V, Granjeaud S, Tagett B, Loriod B, Giaconia A, Jacquemier J *et al.* 2001. c/o Birnbaum D, Inst J Paoli-Calmettes, Lab Biol Tumeurs, FR-13009 Marseille, France. Molecular typing of breast cancer: Transcriptomics and DNA microarrays (French English Abstract). *Bull Cancer* **88:** (3) 277.
Buates S, Matlashewski G*. 2001. *McGill Univ, Dept Microbiol & Immunol, 3775 Univ St, Montreal, Quebec, Canada H3A 2B4. Identification of genes induced by a macrophage activator, S-28463, using gene expression array analysis. *Antimicrob Agents Chemother* **45:** (4) 1137.
Chetcuti A, Margan S, Mann S, Russell P, Handelsman D, Rogers J, Dong QH*. 2001. *Univ Sydney, Dept Med, Sydney, NSW 2006, Australia. Identification of differentially expressed genes in organ-confined prostate cancer by gene expression array. *Prostate* **47:** (2) 132.
Cole J, Tsou R, Wallace K, Gibran N, Isik F*. 2001. *Univ Washington, Med Ctr, Dept Surg, Box 356410, 1959 NE Pacific St, Seattle, Wa 98195, USA. Comparison of normal human skin gene expression using cDNA microarrays. *Wound Repair Regen* **9:** (2) 77.
Cowman AF. 2001. Royal Melbourne Hosp, Walter & Eliza Hall Inst Med Res, Royal Parade, Melbourne, Vic 3050, Australia. Functional analysis of drug resistance in *Plasmodium falciparum* in the post-genomic era. *Int J Parasitol* **31:** (9) 871.
De Backer MD, Ilyina T, Ma XJ, Vandoninck S, Luyten WHML, Van den Bossche H. 2001. RW Johnson Pharmaceut Res Inst, Dept Immunol, 3210 Merryfield Row, San Diego, Ca 92121, USA. Genomic profiling of the response of *Candida albicans* to itraconazole treatment using a DNA microarray. *Antimicrob Agents Chemother* **45:** (6) 1660.
De Veer MJ, Holko M, Frevel M, Walker E, Der S, Paranjape JM, Silverman RH, Williams BRG*. 2001. *Cleveland Clin Fdn, Lerner Res Inst, Dept Canc Biol, 9500 Euclid Ave, Cleveland, Oh 44195, USA. Functional classification of interferon-stimulated genes identified using microarrays. *J Leukoc Biol* **69:** (6) 912.
Dong G, Loukinova E, Chen Z, Gangi L, Chanturita TI, Liu ET, Van Waes C*. 2001. *NIH/Natl Inst Deafness & Commun Disorders, Head & Neck Surg Branch, Bldg 10, Room 5D55, Bethesda, Md 20892, USA. Molecular profiling of transformed and metastatic murine squamous carcinoma cells by differential display and cDNA microarray reveals altered expression of multiple genes related to growth, apoptosis, angiogenesis, and the NF-κB signal pathway. *Cancer Res* **61:** (12) 4797.
El Rifai W, Frierson HF, Harper JC, Powell SM*, Knuutila S. 2001. *Univ Virginia, Dept Med, Box 800708, Charlottesville, Va 22908, USA. Expression profiling of gastric adenocarcinoma using cDNA array. *Int J Cancer* **92:** (6) 832.
Flores-Morales A, Stahlberg N, Tollet-Egnell P, Lundeberg J, Malek RL, Quackenbush J, Lee NH, Norstedt G. 2001. Karolinska Hosp, Dept Mol Med, SE-17176 Stockholm, Sweden. Microarray analysis of the *in vivo* effects of hypophysectomy and growth hormone treatment on gene expression in the rat. *Endocrinology* **142:** (7) 3163.
Geraci MW, Moore M, Gesell T, Yeager ME, Alger L, Golpon H, Gao BF, Loyd JE, Tuder RM, Voelkel NF*. 2001. *Univ Colorado, Hlth Sci Ctr, Div Pulm Sci & Crit Care Med, 4200 East 9th Ave, Denver, Co 80262, USA. Gene expression patterns in the lungs of patients with

Copyright © 2001 John Wiley & Sons, Ltd. 

primary pulmonary hypertension: A gene microarray analysis. *Circ Res* **88:** (6) 555.
Gerhold D, Lu MQ, Xu J, Austin C, Caskey CT, Rushmore T. 2001. Merck Res Labs, Dept Pharmacol, Broad St & Sunneytown Pk, West Point, Pa 19486, USA. Monitoring expression of genes involved in drug metabolism and toxicology using DNA microarrays. *Physiol Genomics* **5:** (4) 161.
Gottlieb B, Beitel LE, Trifiro MA. 2001. Lady Davis Inst Med Res, 3755 Cote Ste Catherine Rd, Montreal, Quebec, Canada. Variable expressivity and mutation databases: The androgen receptor gene mutations database. *Hum Mutat* **17:** (5) 382.
Grunblatt E, Mandel S, Maor G, Youdim MBH*. 2001. *Technion-Israel Inst Technol, Fac Med, Dept Pharm, Eve Top & US Natl Parkinsons Fdn Ctr Neurodegenerat, IL-31096 Haifa, Israel. Gene expression analysis in *N*-methyl-4-phenyl-1,2,3,6-tetrahydropyridine mice model of Parkinson's disease using cDNA microarray: Effect of *R*-apomorphine. *J Neurochem* **78:** (1) 1.
Hui ABY, Lo KW, Yin XL, Poon WS, Ng HK*. 2001. *Chinese Univ, Prince of Wales Hosp, Dept Anat & Cellular Pathol, Shatin, Hong Kong, Peoples Rep China. Detection of multiple gene amplifications in glioblastoma multiforme using array-based comparative genomic hybridization. *Lab Invest* **81:** (5) 717.
Khan J, Wei JS, Ringner M, Saal LH, Ladanyi M, Westermann F, Berthold F, Schwab M, Antonescu CR, Peterson C, Meltzer PS. 2001. NIH/NHGRI, Canc Genet Branch, Bldg 10, Bethesda, Md 20892, USA. Classification and diagnostic prediction of cancers using gene expression profiling and artificial neural networks. *Nat Med* **7:** (6) 673.
Le Naour F, Hohenkirk L, Grolleau A, Misek DE, Lescure P, Geiger JD, Hanash S, Beretta L*. 2001. *Univ Michigan, Dept Microbiol & Immunol, Ann Arbor, Mi 48109, USA. Profiling changes in gene expression during differentiation and maturation of monocyte-derived dendritic cells using both oligonucleotide microarrays and proteomics. *J Biol Chem* **276:** (21) 17920.
Li SY, Ross DT, Kadin ME, Brown PO, Wasik MA*. 2001. *Univ Penn, Med Ctr, Dept Pathol & Lab Med, 3400 Spruce St, Philadelphia, Pa 19104, USA. Comparative genome-scale analysis of gene expression profiles in T-cell lymphoma cells during malignant progression using a complementary DNA microarray. *Am J Pathol* **158:** (4) 1231.
Luzzi V, Holtschlag V, Watson MA*. 2001. *Washington Univ, Sch Med, Dept Pathol & Immunol, Box 8118, 660 Sth Euclid Ave, St Louis, Mo 63110, USA. Expression profiling of ductal carcinoma *in situ* by laser capture microdissection and high-density oligonucleotide arrays. *Am J Pathol* **158:** (6) 2005.
Mohr S, Rihn B. 2001. Inst Natl Rech & Secur, Lab Cancerogenese In vivo, Ave Bourgogne, BP 27, FR-54501 Vandoeuvre-les-Nancy, France. Profiling gene expression in human mesothelioma cells using DNA microarray and high-density filter array technologies (French, English Abstract). *Bull Cancer* **88:** (3) 305.
Raguenez G, Douc-Rasy S, Blanc E, Goldschneider D, Barrois M, Valteau-Couanet D, Benard J*. 2001. *CNRS/UMR 1598, Equipe Neuroblastome Oncogenese & Differenciation, IFR 54, FR-94800 Villejuif, France. A functional gene map is required to adapt therapy of metastatic neuroblastoma (French, English Abstract). *Bull Cancer* **88:** (3) 295.
Sarwal M, Chang S, Barry C, Chen X, Alizadeh A, Salvatierra O, Brown P. 2001. Stanford Univ, Dept Pediat, 703 Welch Rd, Stanford, Ca 94028, USA. Genomic analysis of renal allograft dysfunction using cDNA microarrays. *Transpl Proc* **33:** (1-2) 297.
Theillet C, Orsetti B, Redon R, Du Manoir S. 2001. CRLC, CNRS, Ctr Rech, UMR 5535, Val d'Aurelle-Paul-Lamarque, FR-34298 Montpellier 5, France. Genomic profiling of human tumors: Moving from cytogenetics to DNA arrays (French, English Abstract). *Bull Cancer* **88:** (3) 261.
Villalva C, Trempat P, Zenou RC, Delsol G, Brousset P*. 2001. *CHU Purpan, Anat Pathol Lab, CNRS UPR 2163, Pl Baylac, FR-31059 Toulouse, France. Gene expression profiling in lymphomas by suppression subtractive hybridization (French, English Abstract). *Bull Cancer* **88:** (3) 315.
Zhang HP, Yu CY, Singer B, Xiong MM. 2001. Yale Univ, Sch Med, Dept Epidemiol & Publ Hlth, New Haven, Ct 06520, USA. Recursive partitioning for tumor classification with gene expression microarray data. *Proc Natl Acad Sci U S A* **98:** (12) 6730.
Zhang SL, Day INM, Ye S*. 2001. *Univ Southampton, Southampton Gen Hosp, Human Genet Res Div, Duthie Bldg Mailpoint 808, Tremona Rd, Southampton SO16 6YD, England. Microarrays analysis of nicotine-induced changes in gene expression in endothelial cells. *Physiol Genomics* **5:** (4) 187.
Zohlnhofer D, Richter T, Neumann FJ, Nuhrenberg T, Wessely R, Brandl R, Murr A, Klein CA, Baeuerle PA. 2001. Tech Univ Munich, Deutsch Herzzentrum, Lazerettstr 36, DE-80636 Munich, Germany. Transcriptome analysis reveals a role of interferon-$\gamma$ in human neointima formation. *Mol Cell* **7:** (5) 1059.

### 8 Large-scale mutagenesis programmes

Greco R, Ouwerkerk PBF, Sallaud C, Kohli A, Colombo L, Puigdomenech P, Guiderdoni E, Christou P, Hoge JHC, Pereira A*. 2001. *Plant Res Int, POB 16, NL-6700 AA Wageningen, The Netherlands. Transposon insertional mutagenesis in rice. *Plant Physiol* **125:** (3) 1175.
Ramachandran S, Sundaresan V*. 2001. *Natl Univ Singpore, Inst Mol Agrobiol, 1 Res Link, SG-117604 Singapore, Rep Singapore. Transposons as tools for functional genomics. *Plant Physiol Biochem* **39:** (3-4) 243.

### 9 Functional genomics

Bohnert HJ, Ayoubi P, Borchert C, Bressan RA, Burnap RL, Cushman JC, Cushman MA, Deyholos M, Fischer R, Galbraith DW *et al.* 2001. Univ Arizona, Dept Biochem, Tucson, Az 85721, USA. A genomics approach towards salt stress tolerance. *Plant Physiol Biochem* **39:** (3-4) 295.
Cliften PF, Hillier LW, Fulton L, Graves T, Miner T, Gish WR, Waterston RH, Johnston M*. 2001. *Washington Univ, Sch Med, Dept Genet, St Louis, Mo 63110, USA. Surveying *Saccharomyces* genomes to identify functional elements by comparative DNA sequence analysis. *Genome Res* **11:** (7) 1175.
Frankenberg N, Mukougawa K, Kohchi T, Lagarias JC*. 2001. *Univ Calif, Sect Mol & Cellular Biol, 1 Shields Ave, Davis, Ca 95616, USA. Functional genomic analysis of the HY2 family of ferredoxin-dependent bilin reductases from oxygenic photosynthesis organisms. *Plant Cell* **13:** (4) 965.
Hishigaki H, Nakai K, Ono T, Tanigami A, Takagi T*. 2001. *Univ Tokyo, Inst Med Sci, Human Genome Ctr, Lab Genome Database, 4-6-1 Shirokanedai Minato-ku, Tokyo 108-8639, Japan. Assessment of prediction accuracy of protein function from protein-protein interaction data. *Yeast* **18:** (6) 523.
Ishimaru K, Yano M, Aoki N, Ono K, Hirose T, Lin SY, Monna L, Sasaki T, Ohsugi R. 2001. Natl Inst Agrobiol Resources, Kannondai 2-1-2, Tsukuba, Ibaraki 305 8602, Japan. Toward the mapping of physiological and agronomic characters on a rice function map: QTL analysis and comparison between QTLs and expressed sequence tags. *Theor Appl Genet* **102:** (6-7) 793.
Kan ZY, Rouchka EC, Gish WR, States DJ*. 2001. *Washington Univ, Ctr Computat Biol, St Louis, Mo 63110, USA. Gene structure prediction and alternative splicing analysis using genomically aligned ESTs. *Genome Res* **11:** (5) 889.
Keefe AD, Szostak JW*. 2001. *MGH, Howard Hughes Med Inst, Boston, Ma 02114, USA. Functional proteins from a random-sequence library. *Nature* **410:** (6829) 715.
King RD, Karwath A, Clare A, Dehaspe L. 2001. Univ Wales, Dept Comp Sci, Aberystwyth SY23 3DB, Wales. The utility of different representations of protein sequence for predicting functional class. *Bioinformatics* **17:** (5) 445.
Krogh A, Larsson B, Von Heijne G, Sonnhammer ELL. 2001. Tech Univ Denmark, Ctr Biol Sequence Anal, Bldg 208, DK-2800 Lyngby, Denmark. Predicting transmembrane protein topology with a hidden Markov model: Application to complete genomes. *J Mol Biol* **305:** (3) 567.
Lau GW, Haataja S*, Lonetto M, Kensit SE, Marra A, Bryant AP, McDevitt D, Morrison DA, Holden DW. 2001. *Univ Turku, Dept Med Biochem & Mol Biol, Kiinamyllynkatu 10, FI-20520 Turku, Finland. A functional genomic analysis of type 3 *Streptococcus pneumoniae* virulence. *Mol Microbiol* **40:** (3) 555.
Maeda I, Kohara Y, Yamamoto M, Sugimoto A*. 2001. *Univ Tokyo, Grad Sch Sci, Dept Biophys & Biochem, Bunkyo ku, Tokyo 113 0032, Japan. Large-scale analysis of gene function in *Caenorhabditis elegans* by high-throughput RNAi. *Curr Biol* **11:** (3) 171.
Paustian ML, May BJ, Kapur V*. 2001. *1971 Commonwealth Ave, St Paul, Mn 55108, USA. *Pasteurella multocida* gene expression in response to iron limitation. *Infect Immun* **69:** (6) 4109.

Copyright © 2001 John Wiley & Sons, Ltd. 

Pesaresi P, Varotto C, Richly E, Kurth J, Salamini F, Leister D*. 2001. *Max-Planck-Inst Zuchtungsforsch, ZIGIA, Carl von Linne Weg 10, DE-50829 Cologne, Germany. Functional genomics of *Arabidopsis* photosynthesis. *Plant Physiol Biochem* **39:** (3-4) 285.

Petrash JM, Murthy BSN, Young M, Morris K, Rikimaru L, Griest TA, Harter T. 2001. Washington Univ, Sch Med, Dept Ophthalmol & Visual Sci, 660 Sth Euclid Ave, Campus Box 8096, St Louis, Mo 63110, USA. Functional genomic studies of aldo-keto reductases. *Chem Biol Interact* **130:** (1-3) 673.

Rabitsch KP, Toth A, Galova M, Schleiffer A, Schaffner G, Aigner E, Rupp C, Penkner AM, Moreno-Borchart AC, Primig M *et al.* 2001. c/o Nasmyth K, Res Inst Mol Pathol, Dr Bohr Gasse 7, AU-1030 Vienna, Austria. A screen for genes required for meiosis and spore formation based on whole-genome expression. *Curr Biol* **11:** (13) 1001.

Reymond P. 2001. Univ Lausanne, Inst Ecol, CH-1015 Lausanne, Switzerland. DNA microarrays and plant defence. *Plant Physiol Biochem* **39:** (3-4) 313.

Ruley HE. 2001. Vanderbilt Univ, Sch Med, Dept Microbiol & Immunol, 1161 21st Ave Sth, Nashville, Tn 37232, USA. Functional genomics of the murine immune system. *Immunol Res* **23:** (2-3) 289.

White JA, Benning C*. 2001. *Michigan State Univ, Dept Biochem & Mol Biol, East Lansing, Mi 48824, USA. Genomic approaches towards the engineering of oil seed. *Plant Physiol Biochem* **39:** (3-4) 263.

Wren BW, Linton D, Dorrell N, Karlyshev AV. 2001. London Sch Hyg & Trop Med, Dept Infect & Trop Dis, London WC1E 7HT, England. Post genome analysis of *Campylobacter jejuni*. *J Appl Microbiol* **90:** (Suppl) S36.

Yu JP, Nickels R, McIntosh L*. 2001. *Michigan State Univ, US/DOE, Plant Res Lab, East Lansing, Mi 48824, USA. A genome approach to mitochondrial-nuclear communication in *Arabidopsis*. *Plant Physiol Biochem* **39:** (3-4) 345.

### 10 Transcriptomics

Becker B, Vogt T, Landthaler M, Stolz W*. 2001. *Univ Regensburg, Dept Dermatol, Franz Josef Str Allee 11, DE-93053 Regensburg, Germany. Detection of differentially regulated genes in keratinocytes by cDNA array hybridization: Hsp27 and other novel players in response to artificial ultraviolet radiation. *J Investig Dermatol* **116:** (6) 983.

Blader IJ, Manger ID, Boothroyd JC*. 2001. *Stanford Univ, Dept Microbiol & Immunol, 299 Campus Dr, Stanford, Ca 94305, USA. Microarray analysis reveals previously unknown changes in *Toxoplasma gondii*-infected human cells. *J Biol Chem* **276:** (26) 24223.

Brown AJP, Planta RJ, Restuhadi F, Bailey DA, Butler PR, Cadahia JL, Cerdan ME, De Jonge M, Gardner DCJ, Gent ME, Hayes A, Kolen CP, Lombardia LJ, Murad AM, Oliver RA, Sefton M, Thevelein JM, Tournu H, Van Delft YJ, Verbart DJ, Winderickx J, Oliver SG*. 2001. *Univ Manchester, Sch Biol Sci, 2.205 Stopford Bldg, Oxford Rd, Manchester M13 9PT, England. Transcript analysis of 1003 novel yeast genes using high-throughput northern hybridizations. *EMBO J* **20:** (12) 3177.

He B, Munson AE, Meade BJ*. 2001. *1095 Willowdale Rd, MS 4020, Morgantown, WV 26505, USA. Analysis of gene expression induced by irritant and sensitizing chemicals using oligonucleotide arrays. *Int Immunopharmacol* **1:** (5) 867.

Hihara Y, Kamei A, Kanehisa M, Kaplan A, Ikeuchi M. 2001. Saitama Univ, Fac Sci, Dept Biochem & Mol Biol, Urawa, Saitama 338 8570, Japan. DNA microarray analysis of cyanobacterial gene expression during acclimation to high light. *Plant Cell* **13:** (4) 793.

Hu YJ. 2001. Natl Chiao Tung Univ, Dept Comp & Information Sci, 1001 Ta Hsueh Rd, Hdinchu 300, Taiwan. An integrated approach for genome-wide gene expression analysis. *Comput Methods Program Biomed* **65:** (3) 163.

Hughes TR, Mao M, Jones AR, Burchard J, Marton MJ, Shannon KW, Lefkowitz SM, Ziman M, Schelter JM, Meyer MR *et al.* 2001. c/o Linsley PS, Rosetta Inpharm Inc, 12040 115th Ave NE, Kirkland, Wa 98034, USA. Expression profiling using microarrays fabricated by an ink-jet oligonucleotide synthesizer. *Nat Biotechnol* **19:** (4) 342.

Ideker T, Thorsson V, Siegel AF, Hood LE. 2000. Inst Syst Biol, 4225 Roosevelt Way NE, Suite 200, Seattle, Wa 98105, USA. Testing for differentially-expressed genes by maximum-likelihood analysis for microarray data. *J Comput Biol* **7:** (6) 805.

Kawasaki S, Borchert C, Deyholos M, Wang H, Brazille S, Kawai K, Galbraith D, Bohnert HJ*. 2001. *Univ Arizona, Dept Biochem & Mol Biophys, Tucson, Az 85721, USA. Gene expression profiles during the initial phase of salt stress in rice. *Plant Cell* **13:** (4) 889.

Kerr MK, Martin M, Churchill GA*. 2000. *Jackson Lab, 600 Main St, Bar Harbor, Me 04609, USA. Analysis of variance for gene expression microarray data. *J Comput Biol* **7:** (6) 819.

Leo CP, Pisarska MD, Hsueh AJW*. 2001. *Stanford Univ, Med Ctr, Dept Gynecol & Obstet, Div Reprod Biol, 300 Pasteur Dr, Room A344, Stanford, Ca 94305, USA. DNA array analysis of changes in preovulatory gene expression in the rat ovary. *Biol Reprod* **65:** (1) 269.

Long AD, Mangalam HJ, Chan BYP, Tolleri L, Hatfield GW, Baldi P. 2001. Univ Calif, Sch Biol Sci, Dept Ecol & Evolutionary Biol, Irvine, Ca 92697, USA. Improved statistical inference from DNA microarray data using analysis of variance and a Bayesian statistical framework: Analysis of global gene expression in *Escherichia coli* K12. *J Biol Chem* **276:** (23) 19937.

Munasinghe A, Patankar S, Cook BP, Madden SL, Martin RK, Kyle DE, Shoaibi A, Cummings LM, Wirth DJ*. 2001. *Harvard Univ, Sch Publ Hlth, Dept Immunol & Infect Dis, Bldg 1, Room 704, 665 Huntington Ave, Boston, Ma 02115, USA. Serial analysis of gene expression (SAGE) in *Plasmodium falciparum*: Application of the technique to A-T rich genomes. *Mol Biochem Parasitol* **113:** (1) 23.

Nagasawa Y, Takenaka M*, Kaimori J, Matsuoka Y, Akagi Y, Tsujie M, Imai E, Hori M. 2001. *Osaka Univ, Grad Sch Med 08, Dept Internal Med & Therapeut, 2-2 Yamadaoka Suita, Osaka 560 0871, Japan. Rapid and diverse changes of gene expression in the kidneys of protein-overload proteinuria mice detected by microarray analysis. *Nephrol Dial Transplant* **16:** (5) 923.

Newton MA, Kendziorski CM, Richmond CS, Blattner FR, Tsui KW. 2001. Univ Wisconsin, Dept Stat, 1210 West Dayton St, Madison, Wi 53706, USA. On differential variability of expression ratios: Improving statistical inference about gene expression changes from microarray data. *J Comput Biol* **8:** (1) 37.

Planet PJ, De Salle R, Siddall M, Bael T, Sarkar IN, Stanley SE*. 2001. *Genaissance Pharmaceut, New Haven, Ct 06511, USA. Systematic analysis of DNA microarray data: Ordering and interpreting patterns of gene expression. *Genome Res* **11:** (7) 1149.

Pomposiello PJ, Bennik MHJ, Demple B*. 2001. *Harvard Univ, Sch Publ Hlth, Dept Canc Cell Biol, 665 Huntington Ave, Boston, Ma 02115, USA. Genome-wide transcriptional profiling of the *Escherichia coli* responses to superoxide stress and sodium salicylate. *J Bacteriol* **183:** (13) 3890.

Scandurro AB, Weldon CW, Figueroa YG, Alam J, Beckman BS. 2001. Tulane Hlth Sci Ctr, Dept Microbiol & Immunol, 1430 Tulane Ave, New Orleans, La 70112, USA. Gene microarray analysis reveals a novel hypoxia signal transduction pathway in human hepatocellular carcinoma cells. *Int J Oncol* **19:** (1) 129.

Smith EJ, Shi L, Drummond P, Rodriguez L, Hamilton R, Ramlal S, Smith G, Pierce K, Foster J. 2001. Virginia Polytech Inst & State Univ, 3130 Litton Reaves Hall, Blacksburg, Va 24061, USA. Expressed sequence tags for the chicken genome from a normalized 10-day-old white leghorn whole embryo cDNA library: I. DNA sequence characterization and linkage analysis. *J Hered* **92:** (1) 1.

Thomas JG, OIson JM, Tapscott SJ, Zhao LP*. 2001. *Fred Hutchinson Canc Res Ctr, Div Publ Hlth Sci, Seattle, Wa 98109, USA. An efficient and robust statistical modeling approach to discover differentially expressed genes using genomic expression profiles. *Genome Res* **11:** (7) 1227.

Tusher VG, Tibshirani R, Chu G*. 2001. *Stanford Univ, Dept Med, Ctr Clin Sci Res 1115, 269 Campus Dr, Stanford, Ca 94305, USA. Significance analysis of microarrays applied to the ionizing radiation response. *Proc Natl Acad Sci U S A* **98:** (9) 5116.

Van der Merwe GK, Van Vuuren HJJ, Cooper TG*. 2001. *Univ Tennessee, Dept Microbiol & Immunol, Memphis, Tn 38163, USA. *Cis*-acting sites contributing to expression of divergently transcribed *DAL1* and *DAL4* genes in *S. cerevisiae*: A word of caution when correlating *cis*-acting sequences with genome-wide expression analyses. *Curr Genet* **39:** (3) 156.

Yale J, Bohnert HJ*. 2001. *Univ Arizona, Dept Biochem, Biosci West, East Lowell St, Tucson, Az 85721, USA. Transcript expression in *Saccharomyces cerevisiae* at high salinity. *J Biol Chem* **276:** (19) 15996.

Zhang L, Ma XL, Zhang Q, Ma CL, Wang PP, Sun YF, Zhao YX, Zhang H*. 2001. *Shandong Normal Univ, Dept Biol, Key Lab Plant Stress Res, 88 Wenhua East Rd, CN-250014 Jinan, Peoples Rep China. Expressed sequence tags from a NaCl-treated *Suaeda selsa* cDNA library. *Gene* **267:** (2) 193.

Copyright © 2001 John Wiley & Sons, Ltd. 

### 11 Proteomics

Alaiya AA, Oppermann M, Langridge J, Roblick U, Egevad L, Brandstedt S, Hellstrom M, Linder S, Bergman T, Jornvall H, Auer G*. 2001. *Karolinska Hosp & Inst, Dept Pathol & Oncol, SE-17176 Stockholm, Sweden. Identification of proteins in human prostate tumor material by two-dimensional gel electrophoresis and mass spectrometry. *Cell Mol Life Sci* **58:** (2) 307.

Bell AW, Ward MA, Blackstock WP, Freeman HNM, Choudhary JS, Lewis AP, Chotai D, Fazel A, Gushue JN, Paiement J *et al.* 2001. c/o Bergeron JJM, McGill Univ, Dept Anat & Cells Biol, 3640 Univ St, Montreal, Quebec, Canada H3A 2B2. Proteomics characterization of abundant Golgi membrane proteins. *J Biol Chem* **276:** (7) 5152.

Berggren KN, Chernokalskaya E, Lopez MF, Beechem JM, Patton WF*. 2001. *Mol Probes Inc, Proteomics Sect, 4849 Pitchford Ave, Eugene, Or 97402, USA. Comparison of three different fluorescent visualization strategies for detecting *Escherichia coli* ATP synthase subunits after sodium dodecyl sulfate-polyacrylamide gel electrophoresis. *Proteomics* **1:** (1) 54.

Conrads TP, Alving K, Veenstra TD, Belov ME, Anderson GA, Anderson DJ, Lipton MS, Pasa-Tolic L, Udseth HR, Chrisler WB, Thrall BD, Smith RD*. 2001. *Pacific NW Natl Lab, Mol Biosci Dept, POB 999, Richland, Wa 99352, USA. Quantitative analysis of bacterial and mammalian proteomes using a combination of cysteine affinity tags and $^{15}$N-metabolic labeling. *Anal Chem* **73:** (9) 2132.

Davidsson P, Paulson L, Hesse C, Blennow K, Nilsson CL. 2001. Univ Gothenburg, Sahlgrenska Univ Hosp Molndal, Dept Clin Neurosci, Expt Neurosci Sect, SE-43180 Molndal, Sweden. Proteome studies of human cerebrospinal fluid and brain tissue using a preparative two-dimensional electrophoresis approach prior to mass spectrometry. *Proteomics* **1:** (3) 444.

Derhami K, Zheng J, Li L, Wolfaardt JF, Scott PG*. 2001. *Univ Alberta, Dept Biochem, Edmonton, Alberta, Canada T6G 2H7. Proteomic analysis of human skin fibroblasts grown on titanium: Novel approach to study molecular biocompatibility. *J Biomed Mater Res* **56:** (2) 234.

Duong PT, Chang FN*. 2001. *Temple Univ, Dept Biol, Philadelphia, Pa 19122, USA. A simple method for assigning multiple immunogens to their protein on a two-dimensional blot and its application to asthma-causing allergens. *Electrophoresis* **22:** (10) 2098.

Friso G, Kaiser L, Raud J, Wikstrom L. 2001. Cornell Univ, Dept Plant Biol, 228 Plant Sci Bldg, Ithaca, NY 14853, USA. Differential protein expression in rat trigeminal ganglia during inflammation. *Proteomics* **1:** (3) 397.

Gallardo K, Job C, Groot SPC, Puype M, Demol H, Vandekerckhove J, Job D*. 2001. *Aventis CropSci, Inst Natl Rech Agron-Aventis, Lab Mixte, CNRS, Lyon, France. Proteomic analysis of *Arabidopsis* seed germination and priming. *Plant Physiol* **126:** (2) 835.

Goshe MB, Conrads TP, Panisko EA, Angell NH, Veenstra TD, Smith RD*. 2001. *Pacific NW Natl Lab, Environm & Mol Sci Lab, POB 999, Richland, Wa 99352, USA. Phosphoprotein isotope-coded affinity tag approach for isolating and quantitating phosphopeptides in proteome-wide analyses. *Anal Chem* **73:** (11) 2578.

Grunenfelder B, Rummel G, Vohradsky J, Roder D, Langen H, Jenal U*. 2001. *Univ Basel, Biozentrum, Div Mol Microbiol, Klingelbergstr 70, CH-4056 Basel, Switzerland. Proteomic analysis of the bacterial cell cycle. *Proc Natl Acad Sci U S A* **98:** (8) 4681.

Hampel DJ, Sansome C, Sha M, Brodsky S, Lawson WE, Goligorsky MS*. 2001. *SUNY Stony Brook, Dept Med, Div Nephrol & Hypertension, Hlth Sci Ctr, Stony Brook, NY 11794, USA. Toward proteomics in uroscopy: Urinary protein profiles after radiocontrast medium administration. *J Am Soc Nephrol* **12:** (5) 1026.

Hanson BJ, Schulenberg B, Patton WF, Capaldi RA*. 2001. *Univ Oregon, Inst Mol Biol, Eugene, Or 97403, USA. A novel subfractionation approach for mitochondrial proteins: A three-dimensional mitochondrial proteome map. *Electrophoresis* **22:** (5) 950.

Harrington JJ, Sherf B, Rundlett S, Jackson PD, Perry R, Cain S, Leventhal C, Thornton M, Ramachandran R, Whittington J, *et al.* 2001. Athersys Inc, 3201 Carnegie Ave, Cleveland, Oh 44115, USA. Creation of genome-wide protein expression libraries using random activation of gene expression. *Nat Biotechnol* **19:** (5) 440.

Hirabayashi J, Arata Y, Kasai K. 2001. Teikyo Univ, Fac Pharmaceut Sci, Dept Biol Chem, Sagamiko, Kanagawa 199 019, Japan. Glycome project: Concept, strategy and preliminary application to *Caenorhabditis elegans*. *Proteomics* **1:** (2) 295.

Ito T, Chiba T, Ozawa R, Yoshida M, Hattori M, Sakaki Y. 2001. Kanazawa Univ, Canc Res Inst, Div Genome Biol, 13-1 Takaramachi, Kanazawa, Ishikawa 920 093, Japan. A comprehensive two-hybrid analysis to explore the yeast protein interactome. *Proc Natl Acad Sci U S A* **98:** (8) 4569.

Janulczyk R, Rasmussen M*. 2001. *Lund Univ, Dept Cell & Mol Biol, Sect Mol Pathogenesis, BMC B14, Tornavagen 10, SE-221840 Lund, Sweden. Improved patterns for genome-based screening identifies novel cell wall-attached proteins in Gram-positive bacteria. *Infect Immun* **69:** (6) 4019.

Jurgen B, Hanschke R, Sarvas M, Hecker M, Schweder T*. 2001. *Univ Greifswald, Inst Microbiol, FL Jahnstr 15, DE-17487 Greifswald, Germany. Proteome and transcriptome based analysis of *Bacillus subtilis* cells overproducing an insoluble heterologous protein. *Appl Microbiol Biotechnol* **55:** (3) 326.

Kennedy S. 2001. Oxford Glycoscience UK Ltd, The Forum, 86 Milton Pk, Abingdon OX14 4RY, England. Proteomic profiling from human samples: The body fluid alternative. *Toxicol Lett* **120:** (1-3) 379.

Kim ST, Cho KS, Jang YS, Kang KY*. 2001. *Gyeongsang Natl Univ, Dept Agr Chem, Plant Mol Biol & Biotechnol Res Ctr, Chinju 660701, South Korea. Two-dimensional electrophoretic analysis of rice proteins by polyethylene glycol fractionation for protein arrays. *Electrophoresis* **22:** (10) 2103.

Koc EC, Burkhart W, Blackburn K, Koc H, Moseley A, Spremulli LL*. 2001. *Univ Nth Carolina, Dept Chem, Campus Box 3290, Chapel Hill, NC 27599, USA. Identification of four proteins from the small subunit of the mammalian mitochondrial ribosome using a proteomics approach. *Protein Sci* **10:** (3) 471.

Krapfenbauer K, Berger M, Lubec G, Fountoulakis M*. 2001. *F Hoffmann La Roche Ltd, Pharmaceut Res, Genomics Technol, Bldg 93-444, CH-4070 Basel, Switzerland. Changes in the brain protein levels following administration of kainic acid. *Electrophoresis* **22:** (10) 2086.

Larsen PM, Fey SJ, Larsen MR, Nawrocki A, Andersen HU, Kahler H, Heilmann C, Voss MC, Roepstorff P, Pociot F, Karlsen AE, Nerup J. 2001. Univ Sthn Denmark, Ctr Proteome Anal, Forskerparken, Odense, Denmark. Proteome analysis of interleukin-1β-induced changes in protein expression in rat islets of Langerhans. *Diabetes* **50:** (5) 1056.

Lisacek FC, Traini MD, Sexton D, Harry JL, Wilkins MR. 2001. Univ Versailles St Quentin, Lab Genome & Informatique, 45 Ave Etats Unis, FR-78035 Versailles, France. Strategy for protein isoform identification from expressed sequence tags and its application to peptide mass fingerprinting. *Proteomics* **1:** (2) 186.

Mihr C, Baumgartner M, Dieterich JH, Schmitz UK, Braun HP*. 2001. *Univ Hannover, Abt Angewandte Genet, Herrenhauserstr 2, DE-30419 Hannover, Germany. Proteomic approach for investigation of cytoplasmic male sterility (CMS) in *Brassica*. *J Plant Physiol* **158:** (6) 787.

Paweletz CP, Liotta LA, Petricoin EF*. 2001. *US/FDA, Ctr Biol Evaluat & Res, Div Therapeut Prot, Tissue Proteomics Unit, Bldg 29A, Room 2802, Bethesda, Md 20892, USA. New technologies for biomarker analysis of prostate cancer progression: Laser capture microdissection and tissue proteomics. *Urology* **57:** (4A Suppl) 160.

Perrot F, Hebraud M, Charlionet R, Junter GA, Jouenne T*. 2001. *Fac Sci, CNRS UMR 6522, FR-76821 Mont St Aignan, France. Cell immobilization induces change in the protein response of *Escherichia coli* K-12 to a cold shock. *Electrophoresis* **22:** (10) 2110.

Saleh MT, Fillon M, Brennan PJ, Belisle JT*. 2001. *Colorado State Univ, Dept Microbiol, Mycobacteria Res Labs, Fort Collins, Co 80523, USA. Identification of putative exported/secreted proteins in prokaryotic proteomes. *Gene* **269:** (1-2) 195.

Schrimpf SP, Langen H, Gomes AV, Wahlestedt C*. 2001. *Karolinska Inst, Ctr Genomics Res, Berzellius Vag 37, SE-17177 Stockholm, Sweden. A two-dimensional protein map of *Caenorhabditis elegans*. *Electrophoresis* **22:** (6) 1224.

Schwartz R, Ting CS, King J. 2001. MIT, Dept Biol, 77 Massachusetts Ave, Cambridge, Ma 02139, USA. Whole proteome pI values correlate with subcellular localizations of proteins for organisms within the three domains of life. *Genome Res* **11:** (5) 703.

Singh VK, Jayaswal RK, Wilkinson BJ*. 2001. *Illinois State Univ, Dept Sci Biol, Microbiol Grp, Normal, Il 61790, USA. Cell wall-active antibiotic induced proteins of *Staphylococcus aureus* identified using a proteomic approach. *FEMS Microbiol Lett* **199:** (1) 79.

Spahr CS, Davis MT, McGinley MD, Robinson JH, Bures EJ, Beierle J, Mort J, Courchesne PL, Chen K, Wahl RC, *et al.* 2001. c/o Patterson SD, Celera Genomics, 45 West Gude Dr, Rockville, Md 20850, USA.

Copyright © 2001 John Wiley & Sons, Ltd. 

Towards defining the urinary proteome using liquid chromatography-tandem mass spectrometry I. Profiling an unfractionated tryptic digest. *Proteomics* **1:** (1) 93.

Stevanovic S, Bohley P. 2001. Univ Tubingen, Inst Zellbiol, Immunol Abt, DE-72076 Tubingen, Germany. Proteome analysis by three-dimensional protein separation: Turnover of cytosolic proteins in hepatocytes. *Biol Chem* **382:** (4) 677.

Tonella L, Hoogland C, Binz PA, Appel RD, Hochstrasser DF, Sanchez JC. 2001. Univ Hosp Geneva, Cent Lab Clin Chem, 24 rue Micheli Crest, CH-1211 Geneva 14, Switzerland. New perspectives in the *Escherichia coli* proteome investigation. *Proteomics* **1:** (3) 409.

Vandahl BB, Birkelund S, Demol H, Hoorelbeke B, Christiansen G, Vandekerckhove J, Gevaert K. 2001. Univ Aarhus, Dept Med Microbiol & Immunol, Bartholin Bldg, DK-8000 Aarhus C, Denmark. Proteome analysis of the *Chlamydia pneumoniae* elementary body. *Electrophoresis* **22:** (6) 1204.

Vlahou A, Schellhamrner PF, Mendrinos S, Patel K, Kondylis FI, Gong L, Nasim S, Wright GL. 2001. Eastern Virginia Med Sch, Dept Microbiol & Mol Cell Biol, 700 West Olney Rd, Norfolk, Va 23507, USA. Development of a novel proteomic approach for the detection of transitional cell carcinoma of the bladder in urine. *Am J Pathol* **158:** (4) 1491.

Watkins B, Szaro R, Ball S, Knubovets T, Briggman J, Hlavaty JJ, Kusinitz F, Stieg A, Wu YJ*. 2001. *Matritech Res, 330 Nevada St, Newton, Ma 02460, USA. Detection of early-stage cancer by serum protein analysis. *Am Lab* **33:** (12) 32.

Yan JX, Harry RA, Wait R, Welson SY, Emery PW, Preedy VR, Dunn MJ. 2001. Amersham Pharmacia Biotech, Amersham Labs, White Lion Rd, Amersham HP7 9LL, England. Separation and identification of rat skeletal muscle proteins using two-dimensional gel electrophoresis and mass spectrometry. *Proteomics* **1:** (3) 424.

Yao XD, Freas A, Ramirez J, Demirev PA, Fenselau C*. 2001. *Univ Maryland, Dept Chem & Biochem, College Park, Md 20742, USA. Proteolytic $^{18}O$ labeling for comparative proteomics: Model studies with two serotypes of adenovirus. *Anal Chem* **73:** (13) 2836.

Zhang HT, Kacharmina JE, Miyashiro K, Greene MI*, Eberwine J. 2001. *Univ Penn, Abramson Inst Canc Res, Dept Pathol, Philadelphia, Pa 19104, USA. Protein quantification from complex protein mixtures using a proteomics methodology with single-cell resolution. *Proc Natl Acad Sci U S A* **98:** (10) 5497.

Zucchi I, Bini L, Valaperta R, Ginestra A, Albani D, Susani L, Sanchez JC, Liberatori S, Magi B, Raggiaschi R, *et al.* 2001. CNR, Ist Tecnol Biomed Avanzate, via F Ili Cervi 93, IT-20090 Milan, Italy. Proteomic dissection of dome formation in a mammary cell line: Role of tropomyosin-5b and maspin. *Proc Natl Acad Sci U S A* **98:** (10) 5608.

### 12 Protein structural genomics

Balaji S, Srinivasan N*. 2001. *Indian Inst Sci, Mol Biophys Unit, IN-560012 Bangalore, India. Use of a database of structural alignments and phylogenetic trees in investigating the relationship between sequence and structural variability among homologous proteins. *Protein Eng* **14:** (4) 219.

Bladon P. 2001. Interprobe Chem Serv, Gallowhill House, Larch Ave, Glasgow G66 4HX, Scotland. A simple method for aligning many protein sequences. *J Chem Inform Comput Sci* **41:** (2) 278.

Bujnicki JM, Elofsson A, Fischer D, Rychlewski L. 2001. Int Inst Mol & Cell Biol, Bioinformatics Lab, ul KS Trojdena 4, PL-02109 Warsaw, Poland. LiveBench-1: Continuous benchmarking of protein structure prediction servers. *Protein Sci* **10:** (2) 352.

Ding CHQ, Dubchak I. 2001. Univ Calif, Lawrence Berkeley Natl Lab, NERSC Div, Berkeley, Ca 94720, USA. Multi-class protein fold recognition using support vector machines and neural networks. *Bioinformatics* **17:** (4) 349.

Eisenhaber B, Bork P, Eisenhaber F. 2001. Res Inst Mol Pathol, Dr Bohr Gasse 7, AU-1030 Vienna, Austria. Post-translational GPI lipid anchor modification of proteins in kingdoms of life: Analysis of protein sequence data from complete genomes. *Protein Eng* **14:** (1) 17.

Fetrow JS, Siew N, Di Gennaro JA, Martinez-Yamout M, Dyson HJ, Skolnick J*. 2001. *Donald Danforth Plant Sci Ctr, 893 Nth Warson Rd, St Louis, Mo 63141, USA. Genomic-scale comparison of sequence- and structure-based methods of function prediction: Does structure provide additional insight? *Protein Sci* **10:** (5) 1005.

Garian R. 2001. George Mason Univ, Sch Comp Sci, Fairfax, Va 22030, USA. Prediction of quaternary structure from primary structure. *Bioinformatics* **17:** (6) 551.

Gille C, Frommel C. 2001. Humboldt Univ, Fac Med, Inst Biochem Charite, Monbijoustr 2A, DE-10117 Berlin, Germany. STRAP: Editor for STRuctural Alignments of Proteins. *Bioinformatics* **17:** (4) 377.

Jennings AJ, Edge CM, Sternberg MJE. 2001. SmithKline Beecham Pharmaceut, Discovery Chem, New Frontiers Sci Pk, 3rd Ave, Harlow CM19 5AW, England. An approach to improving multiple alignments of protein sequences using predicted secondary structure. *Protein Eng* **14:** (4) 227.

Looger LL, Hellinga HW*. 2001. *Duke Univ, Med Ctr, Dept Biochem, Box 3711, Durham, NC 27710, USA. Generalized dead-end elimination make large-scale protein side-chain structure prediction tractable: Implications for protein design and structural genomics. *J Mol Biol* **307:** (1) 429.

May ACW. 2001. Natl Inst Med Res, Div Math Biol, Mill Hill, London NW7 1AA, England. Optimal classification of protein sequences and selection of representative sets from multiple alignments: Application to homologous families and lessons for structural genomics. *Protein Eng* **14:** (4) 209.

Sim KL, Uchida T, Miyano S. 2001. Univ Kentucky, Dept Biochem, Kentucky Ctr Struct Biol, 800 Rose St, Lexington, Ky 40536, USA. ProDDO: A database of disordered proteins from the Protein Data Bank (PDB). *Bioinformatics* **17:** (4) 379.

### 13 Metabolomics

De Vienne D, Bost B, Fievet J, Zivy M, Dillmann C. 2001. INRA/INA-PG/UPS, Stn Genet Vegetale, Ferme Moulon, FR-91190 Gif-sur-Yvette, France. Genetic variability of proteome expression and metabolic control. *Plant Physiol Biochem* **39:** (3-4) 271.

Ideker T, Thorsson V, Ranish JA, Christmas R, Buhler J, Eng JK, Bumgarner R, Goodlett DR, Aebersold R, Hood L. 2001. Inst Syst Biol, 4225 Roosevelt Way NE, Suite 200, Seattle, Wa 98105, USA. Integrated genomic and proteomic analyses of a systematically perturbed metabolic network. *Science* **292:** (5518) 929.

Smulski DR, Huang LXL, McCluskey MP, Reeve MJG, Vollmer AC, Van Dyk TK, La Rossa RA*. 2001. *DuPont Co Inc, Cent Res & Dev, Expt Stn, Biochem Sci & Engn, POB 80173, Wilmington, De 19880, USA. Combined, functional genomic-biochemical approach to intermediary metabolism: Interaction of acivicin, a glutamine amidotransferase inhibitor, with *Escherichia coli* K-12. *J Bacteriol* **183:** (11) 3353.

Stancato LF, Petricoin EF*. 2001. *US/FDA, Ctr Biol Evaluat & Res, Tissue Proteomics Unit, Div Therapeut Prod, Bethesda, Md, USA. Fingerprinting of signal transduction pathways using a combination of anti-phosphotyrosine immunoprecipitations and two-dimensional polyacrylamide gel electrophoresis. *Electrophoresis* **22:** (10) 2120.

Yaffe MB, Leparc GG, Lai J, Obata T, Volinia S, Cantley LC. 2001. Harvard Univ, Inst Med, Dept Med, Div Signal Transduction, 10th Floor, 330 Brookline Ave, Boston, Ma 02215, USA. A motif-based profile scanning approach for genome-wide prediction of signalling pathways. *Nat Biotechnol* **19:** (4) 348.

### 14 Genomic approaches to development

Chen JN, Van Bebber F, Goldstein AM, Serluca FC, Jackson D, Childs S, Serbedzija G, Warren KS, Mably JD, Lindahl P, Mayer A, Haffter P, Fishman MC*. 2001. *Massachusetts Gen Hosp, Cardiovasc Res Ctr, 149 13th St, Charlestown, Ma 02129, USA. Genetic steps to organ laterality in zebrafish. *Comp Funct Genom* **2:** (2) 60.

Christiansen JH, Coles EG, Robinson V, Pasini A, Wilkinson DG*. 2001. *Natl Inst Med Res, Div Dev Neurobiol, Mill Hill, London NW7 1AA, England. Screening from a subtracted embryonic chick hindbrain cDNA library: Identification of genes expressed during hindbrain, midbrain and cranial neural crest development. *Mech Dev* **102:** (1-2) 119.

Kuwabara PE, O'Neil N. 2001. Sanger Ctr, Wellcome Trust Genome Campus, Hinxton CB10 1SA, England. The use of functional genomics in *C. elegans* for studying human development and disease. *J Inherit Metab Dis* **24:** (2) 127.

Liu HC, He ZM, Rosenwaks Z. 2001. Cornell Univ, Weill Med Coll, Ctr Reprod Med & Infertil, 515 East 71st St, New York, NY 10021, USA. Application of complementary DNA microarray (DNA chip) technology in the study of gene expression profiles during folliculogenesis. *Fertil Steril* **75:** (5) 947.

Copyright © 2001 John Wiley & Sons, Ltd. 

Shen MM. 2001. Univ Med & Dent New Jersey, CABM, Robert Wood Johnson Med Sch, 679 Hoes Lane, Piscataway, NJ 08854, USA. Identification of differentially expressed genes in mouse development using differential display and *in situ* hybridization. *Methods* **24:** (1) 15.

Zhu T, Budworth P, Han B, Brown D, Chang HS, Zou GZ, Wang X. 2001. Torrey Mesa Res Inst Inc, 3115 Merryfield Row, San Diego, Ca 92121, USA. Toward elucidating the global gene expression patterns of developing *Arabidopsis*: Parallel analysis of 8300 genes by a high-density oligonucleotide probe array. *Plant Physiol Biochem* **39:** (3-4) 221.

### 15 Technological advances

Beekman M, Lakenburg N, Cherny SS, De Knijff P, Kluft CC, Van Ommen GJB, Vogler GP, Frants RR, Boomsma DI, Slagboom PE*. 2001. *TNO Prevent & Hlth, Gaubus Lab, POB 2215, NL-2301 CE Leiden, The Netherlands. A powerful and rapid approach to human genome scanning using small quantities of genomic DNA. *Genet Res* **77:** (2) 129.

Belosludtsev Y, Iverson B, Lemeshko S, Eggers R, Wiese R, Lee S, Powdrill T, Hogan M. 2001. Genometrix Inc, 3608 Res Forest Dr, Suite B7, The Woodlands, Tx 77381, USA. DNA microarrays based on noncovalent oligonucleotide attachment and hybridization in two dimensions. *Anal Biochem* **292:** (2) 250.

Bochner BR, Gadzinski P, Panomitros E. 2001. Biolog Inc, Hayward, Ca 94545, USA. Phenotype MicroArrays for high-throughput phenotypic testing and assay of gene function. *Genome Res* **11:** (7) 1246.

Butt A, Davison MD, Smith GJ, Young JA, Gaskell SJ, Oliver SG*, Beynon RJ. 2001. *Univ Manchester, Sch Biol Sci, Room 2.205 Stopford Bldg, Oxford Rd, Manchester M13 9PT, England. Chromatographic separations as a prelude to two-dimensional electrophoresis in proteomics analysis. *Proteomics* **1:** (1) 42.

Castagnoli L, Zucconi A, Quondam M, Rossi M, Vaccaro P, Panni S, Santonico E, Dente L, Cesareni G*. 2001. *Univ Roma Tor Vergata, Dept Biol, via Ricerca Scientif, IT-00133 Rome, Italy. Alternative bacteriophage display systems. *Comb Chem High Throughput Scr* **4:** (2) 121.

Conrad CC, Malakowsky CA, Talent J, Rong D, Lakdawala S, Gracy RW*. 2001. *Univ Nth Texas, Ctr Hlth Sci, Dept Mol Biol & Immunol, Mol Aging Unit, Fort Worth, Tx 76107, USA. Chemiluminescent standards for quantitative comparison of two-dimensional electrophoresis western blots. *Proteomics* **1:** (3) 365.

Crameri R, Kodzius R. 2001. Swiss Inst Allergy & Asthma Res, SIAF, Obere Str 22, CH-7270 Davos, Switzerland. The powerful combination of phage surface display of cDNA libraries and high throughput screening. *Comb Chem High Throughput Scr* **4:** (2) 145.

Davis MT, Spahr CS, McGinley MD, Robinson JH, Bures EJ, Beierle J, Mort J, Yu W, Luethy R, Patterson SD*. 2001. *Celera Genomics, 45 West Gude Dr, Rockville, Md 20850, USA. Towards defining the urinary proteome using liquid chromatography-tandem mass spectrometry II. Limitations of complex mixture analyses. *Proteomics* **1:** (1) 108.

Figeys D, McBroom LD, Moran MF*. 2001. *MDS Proteomics Inc, 480 Univ Ave, Toronto, Ontario, Canada M5G 1V2. Mass spectrometry for the study of protein-protein interactions. *Methods* **24:** (3) 230.

Galvani M, Hamdan M, Herbert B, Righetti PG*. 2001. *Univ Verona, Dept Agr & Ind Biotechnol, Strada Grazie 15, IT-37134 Verona, Italy. Alkylation kinetics of proteins in preparation for two-dimensional maps: A matrix assisted laser desorption/ionization-mass spectrometry investigation. *Electrophoresis* **22:** (10) 2058.

Galvani M, Rovatti L, Hamdan M, Herbert B, Righetti PG*. 2001. *Address as above. Protein alkylation in the presence/absence of thiourea in proteome analysis: A matrix-assisted laser desorption/ionization-time of flight-mass spectrometry investigation. *Electrophoresis* **22:** (10) 2066.

Hansson M, Samuelson P, Gunneriusson E, Stahl S*. 2001. *Royal Inst Technol, KTU, Dept Biotechnol, SE-10044 Stockholm, Sweden. Surface display on Gram positive bacteria. *Comb Chem High Throughput Scr* **4:** (2) 171.

Herbert B, Galvani M, Hamdan M, Olivieri E, MacCarthy J, Pedersen S, Righetti PG*. 2001. *Univ Verona, Dept Agr & Ind Biotechnol, Strada Grazie 15, IT-37134 Verona, Italy. Reduction and alkylation of proteins in preparation of two-dimensional map analysis: Why, when, and how? *Electrophoresis* **22:** (10) 2046.

Hwang UW, Park CJ, Yong TS, Kim W*. 2001. *Seoul Natl Univ, Coll Nat Sci, Sch Biol Sci, Seoul 151742, South Korea. One-step PCR amplification of complete arthropod mitochondrial genomes. *Mol Phylogenet Evol* **19:** (3) 345.

Jacobsson K, Frykberg L. 2001. Swedish Univ Agr Sci, Dept Microbiol, Box 705, SE-75007 Uppsala, Sweden. Shotgun phage display cloning. *Comb Chem High Throughput Scr* **4:** (2) 135.

Jiang GF, Chen RS, Yan H, Qi QY. 2001. Beijing Univ, Dept Phys, CN-100871 Beijing, Peoples Rep China. A new method of preparing fiber-optic DNA biosensor and its array for gene detection. *Sci China C Life Sci* **44:** (1) 33.

Krin E, Hommais F*, Soutourina O, Ngo S, Danchin A, Bertin P. 2001. *Inst Pasteur, Unite Genet Genomes Bacteriens, 28 rue Dr Roux, FR-75724 Paris 15, France. Description and application of a rapid method for genomic DNA direct sequencing. *FEMS Microbiol Lett* **199:** (2) 229.

Larsen MR, Sorensen GL, Fey SJ, Larsen PM, Roepstorff P*. 2001. *Odense Univ, Univ Sthn Denmark, Dept Biochem & Mol Biol, Campusvej 55, DK-5230 Odense M, Denmark. Phospho-proteomics: Evaluation of the use of enzymatic de-phosphorylation and differential mass spectrometric peptide mass mapping for site specific phosphorylation assignment in proteins separated by gel electrophoresis. *Proteomics* **1:** (2) 223.

Lazo GR, Tong J, Miller R, Hsia C, Rausch C, Kang Y, Anderson OD. 2001. USDA/ARS, Western Reg Res Ctr, 800 Buchanan St, Albany, Ca 94710, USA. Software scripts for quality checking of high-throughput nucleic acid sequencers. *Biotechniques* **30:** (6) 1300.

Le Bihan T, Pinto D, Figeys D*. 2001. *MDS-Ocata, 480 Univ Ave, Suite 401, 4th Floor, Toronto, Ontario, Canada M5G 1V2. Nanoflow gradient generator coupled with $\mu$-LC-ESI-MS/MS for protein identification. *Anal Chem* **73:** (6) 1307.

Luker GD, Piwnica-Worms D*. 2001. *Washington Univ, Sch Med, Edward Mallinckrodt Inst Radiol, Mol Imaging Ctr, Lab Mol Radiopharmacol, St Louis, Mo 63110, USA. Beyond the genome: Molecular imaging *in vivo* with PET and SPECT. *Acad Radiol* **8:** (1) 4.

Mahon P, Dupree P*. 2001. *Univ Cambridge, Dept Biochem, Bldg O, Downing Site, Cambridge CB2 1QW, England. Quantitative and reproducible two-dimensional gel analysis using Phoretix 2D Full. *Electrophoresis* **22:** (10) 2075.

Malone JP, Radabaugh MR, Leimgruber RM, Gerstenecker GS. 2001. Pharmacia Corp, 700 Chesterfield Pkwy Nth, St Louis, Mo 63198, USA. Practical aspects of fluorescent staining for proteomic applications. *Electrophoresis* **22:** (5) 919.

Mann M, Pandey A. 2001. Univ Sthn Denmark, Ctr Expt Bioinformatics, PIL, Campusvej 55, DK-5230 Odense M, Denmark. Use of mass spectrometry-derived data to annotate nucleotide and protein sequence databases. *Trends Biochem Sci* **26:** (1) 54.

Medico E, Gambarotta G, Gentile A, Comoglio PM, Soriano P. 2001. Univ Turin, Sch Med, Inst Canc Res & Treatment, IT-10060 Candiolo, Italy. A gene trap vector system for identifying transcriptionally responsive genes. *Nat Biotechnol* **19:** (6) 579.

Muller DR, Schindler P, Towbin H, Wirth U, Voshol H, Hoving S, Steinmetz MO. 2001. Novartis Pharma AG, Functional Genomics, CH-4002 Basel, Switzerland. Isotope tagged cross linking reagents. A new tool in mass spectrometric protein interaction analysis. *Anal Chem* **73:** (9) 1927.

Oda Y, Nagasu T, Chait BT*. 2001. *Rockefeller Univ, 1230 York Ave, New York, NY 10021, USA. Enrichment analysis of phosphorylated proteins as a tool for probing phosphoproteome. *Nat Biotechnol* **19:** (4) 379.

Premstaller A, Oberacher H, Walcher W, Timperio AM, Zolla L, Chervet JP, Cavusoglu N, Van Dorsselaer A, Huber CG*. 2001. *Innsbruck Univ, Inst Analyt Chem & Radiochem, Innrain 52A, AU-6020 Innsbruck, Austria. High-performance liquid chromatography-electrospray ionization mass spectrometry using monolithic capillary columns for proteomic studies. *Anal Chem* **73:** (11) 2390.

Reid R, Dix DJ, Miller D, Krawetz SA*. 2001. *Wayne State Univ, Sch Med, Dept Obstet & Gynecol, 275 East Hancock, Detroit, Mi 48201, USA. Recovering filter-based microarray data for pathways analysis using a multipoint alignment strategy. *Biotechniques* **30:** (4) 762.

Rudd PM, Colominas C, Royle L, Murphy N, Hart E, Merry AH, Hebestreit HF, Dwek RA. 2001. Univ Oxford, Dept Biochem, Glycobiol Inst, Sth Parks Rd, Oxford OX1 3QU, England. A high-performance liquid chromatography based strategy for rapid, sensitive sequencing of *N*-linked oligosaccharide modifications to proteins in sodium dodecyl sulphate polyacrylamide electrophoresis gel bands. *Proteomics* **1:** (2) 285.

Copyright © 2001 John Wiley & Sons, Ltd.

Shen YF, Zhao R, Belov ME, Conrads TP, Anderson GA, Tang KQ, Pasa-Tolic L, Veenstra TD, Lipton MS, Udseth HR, Smith RD*. 2001. *Pacific NW Natl Labs, Environm Mol Sci Lab, Richland, Wa 99352, USA. Packed capillary reversed-phase liquid chromatography with high-performance electrospray ionization Fourier transform ion cyclotron resonance mass spectrometry for proteomics. *Anal Chem* **73:** (8) 1766.

Shen YF, Tolic N, Zhao R, Pasa-Tolic L, Li LJ, Berger SJ, Harkewicz R, Anderson GA, Belov ME, Smith RD*. 2001. *Address as above. High-throughput proteomics using high efficiency multiple-capillary liquid chromatography with on-line high-performance ESI FTICR mass spectrometry. *Anal Chem* **73:** (13) 3011.

Shevchenko A, Sunyaev S, Loboda A, Shevehenko A, Bork P, Ens P, Standing KG. 2001. MPI Mol Cell Biol & Genet, DE-01307 Dresden, Germany. Charting the proteomes of organisms with unsequenced genomes by MALDI-quadrupole time of flight mass spectrometry and BLAST homology searching. *Anal Chem* **73:** (9) 1917.

Shevchenko A, Loboda A, Ens W, Schraven B, Standing KG, Shevchenko A. 2001. European Mol Biol Lab, Peptide & Prot Grp, Meyerhofstr 1, DE-69012 Heidelberg, Germany. Archived polyacrylamide gels as a resource for proteome characterization by mass spectrometry. *Electrophoresis* **22:** (6) 1194.

Stensballe A, Andersen S, Jensen ON*. 2001. *Odense Univ, Univ Sthn Denmark, Dept Biochem & Mol Biol, Campusvej 55, DK-5230 Odense M, Denmark. Characterization of phosphoproteins from electrophoretic gels by nanoscale Fe(III) affinity chromatography with off-line mass spectrometry analysis. *Proteomics* **1:** (2) 207.

Stoeckli M, Chaurand P, Hallahan DE, Caprioli RM*. 2001. *Vanderbilt Univ, Sch Med, Mass Spectrometry Res Ctr, Nashville, Tn 37212, USA. Imaging mass spectrometry: A new technology for the analysis of protein expression in mammalian tissues. *Nat Med* **7:** (4) 493.

Taton TA, Lu G, Mirkin CA*. 2001. *Northwestern Univ, Dept Chem, 2145 Sheridan Rd, Evanston, Il 60208, USA. Two-color labeling of oligonucleotide arrays *via* size-selective scattering of nanoparticle probes. *J Am Chem Soc* **123:** (21) 5164.

Tonge R, Shaw J, Middleton B, Rowlinson R, Rayner S, Young J, Pognan F, Hawkins E, Currie I, Davison M. 2001. AstraZeneca Pharmaceut, Proteomics Grp, Enabling Sci & Technol Biol, Alderley Pk, Macclesfield SK10 7TG, England. Validation and development of fluorescence two-dimensional differential gel electrophoresis proteomics technology. *Proteomics* **1:** (3) 377.

Tseng GC, Oh MK, Rohlin L, Liao JC, Wong WH*. 2001. *Harvard Univ, Sch Publ Hlth, Dept Biostat, 655 Huntington Ave, Boston, Ma 02115, USA. Issues in cDNA microarray analysis: Quality filtering, channel normalization, models of variations and assessment of gene effects. *Nucleic Acids Res* **29:** (12) 2549.

Twerenbold D, Gerber D, Gritti D, Gonin Y, Netuschill A, Rossel F, Schenker D, Vuilleumier JL. 2001. Univ Neuchatel, Inst Phys, rue A-L Breguet 1, CH-2000 Neuchatel, Switzerland. Single molecule detector for mass spectrometry with mass independent detection efficiency. *Proteomics* **1:** (1) 66.

Verhaert P, Uttenweiler-Joseph S, De Vries M, Loboda A, Ens W, Standing KG. 2001. NV Organon, Analyt Chem Res, Bio(Macro) MolecMass Spect Grp, Molenstr 110, RK 1219, POB 20, NL-5340 BH Oss, The Netherlands. Matrix-assisted laser desorption/ionization quadrupole time-of-flight mass spectrometry: An elegant tool for peptidomics. *Proteomics* **1:** (1) 118.

Vogel JS, Grant PG, Buchholz BA, Dingley K, Turteltaub KW. 2001. Lawrence Livermore Natl Lab, Ctr Accelerator Mass Spectrometry, 7000 East Ave, Livermore, Ca 94551, USA. Attomole quantitation of protein separations with accelerator mass spectrometry. *Electrophoresis* **22:** (10) 2037.

Wall DB, Lubman DM*. 2001. *Univ Michigan, Dept Chem, 930 Nth Univ, Ann Arbor, Mi 48109, USA. 2-D liquid separations of proteins from cancer cell lysates. *Am Lab* **33:** (12) 13.

Weitzhandler M, Farnan D, Rohrer JS, Avdalovic N. 2001. Dionex Chem Corp, 445 Lakeside Dr, Sunnyvale, Ca 94086, USA. Protein variant separations using cation exchange chromatography on grafted, polymeric stationary phases. *Proteomics* **1:** (2) 179.

Westbrook JA, Yan JX, Wait R, Dunn MJ. 2001. Harefield Hosp, Imperial Coll, Sch Med, Heart Sci Ctr, Natl Heart & Lung Inst, Harefield UB9 6JH, England. A combined radiolabelling and silver staining technique for improved visualisation, localisation and identification of proteins separated by two-dimensional gel electrophoresis. *Proteomics* **1:** (3) 370.

Yamagaki T, Nakanishi H. 2001. Univ Tokyo, Dept Chem, Sch Sci, Bunkyo ku, 7-3-1 Hongo, Tokyo 113 0033, Japan. Ion intensity analysis of post-source decay fragmentation in curved-field reflectron matrix-assisted laser desorption/ionization time-of-flight mass spectrometry of carbohydrates: For structural characterization of glycosylation in proteome analysis. *Proteomics* **1:** (2) 329.

Zarrinkar PP, Mainquist JK, Zamora M, Stern D, Welsh JB, Sapinoso LM, Hampton GM, Lockhart DJ. 2001. Novartis Res Fdn, Genomics Inst, San Diego, Ca 92121, USA. Arrays of arrays for high-throughput gene expression profiling. *Genome Res* **11:** (7) 1256.

Zhu YY, Machleder EM, Chenchik A, Li R, Siebert PD*. 2001. *Clontech Labs Inc, 1020 East Meadow Circle, Palo Alto, Ca 94303, USA. Reverse transcriptase template switching: A SMART™ approach for full-length cDNA library construction. *Biotechniques* **30:** (4) 892.

### 16 Bioinformatics

Aach J, Church GM*. 2001. *Harvard Univ, Sch Med, Dept Genet, 200 Longwood Ave, Boston, Ma 02115, USA. Aligning gene expression time series with time wrapping algorithms. *Bioinformatics* **17:** (6) 495.

Almeida JS, Carrico JA, Maretzek A, Noble PA, Fletcher M. 2001. Univ Nova Losboa, Inst Tecnol Quim & Biol, POB 127, PT-2780 Oeiras, Portugal. Analysis of genomic sequences by Chaos Game Representation. *Bioinformatics* **17:** (5) 429.

Apweiler R, Kersey P, Junker V, Bairoch A. 2001. Eur Bioinformatics Inst, EMBL Outstn, Wellcome Trust Genome Campus, Cambridge CB10 1SD, England. Technical comment to "Database verification studies of SWISS-PROT and GenBank" by Karp *et al. Bioinformatics* **17:** (6) 533.

Arslan AN, Egecioglu O, Pevzner PA. 2001. Univ Calif, Dept Comp Sci, Santa Barbara, Ca 93106, USA. A new approach to sequence comparison: Normalised sequence alignment. *Bioinformatics* **17:** (4) 327.

Baldi P, Long AD. 2001. Univ Calif, Dept Information & Comput Sci, Irvine, Ca 92697, USA. A Bayesian framework for the analysis of microarray expression data: Regularized *t*-test and statistical inferences of gene changes. *Bioinformatics* **17:** (6) 509.

Basu MK. 2001. Ctr Cellular & Mol Biol, IN-500007 Hyderabad, India. SeWeR: A customizable and integrated dynamic HTML interface to bioinformatics services. *Bioinformatics* **17:** (6) 577.

Besemer J, Lomsadze A, Borodovsky M*. 2001. *Georgia Inst Technol, Sch Biol, Atlanta, Ga 30332, USA. GeneMarkS: A self-training method for prediction of gene starts in microbial genomes. Implications for finding sequence motifs in regulatory regions. *Nucleic Acids Res* **29:** (12) 2607.

Biswas M, Kanapin A, Apweiler R. 2001. Eur Bioinformatics Inst, EMBL Outstn, Wellcome Trust Genome Campus, Cambridge CB10 1SD, England. Application of InterPro for the functional classification of the proteins of fish origin in SWISS-PROT and TrEMBL. *J Biosci* **26:** (2) 277.

Brinkman FSL, Wan I, Hancock REW, Rose AM, Jones SJ*. 2001. *British Columbia Canc Agcy, Genome Sequence Ctr, Vancouver, Brit Columbia, Canada V5Z 4E6. PhyloBLAST: Facilitating phylogenetic analysis of BLAST results. *Bioinformatics* **17:** (4) 385.

Buhler J. 2001. Univ Washington, Dept Comp Sci & Engn, Seattle, Wa 98195, USA. Efficient large-scale sequence comparison by locality-sensitive hashing. *Bioinformatics* **17:** (5) 419.

Bukhman YV, Skolnick J*. 2001. *Donald Danforth Plant Sci Ctr, Lab Computat Genomics, 893 Nth Warson Rd, St Louis, Mo 63141, USA. BioMolQuest: Integrated database-based retrieval of protein structural and functional information. *Bioinformatics* **17:** (5) 468.

Bushel PR, Hamadeh H, Bennett L, Sieber S, Martin K, Nuwaysir EF, Johnson K, Reynolds K, Paules RS, Afshari CA*. 2001. *NIEHS, Mol Carcinogenesis Lab, POB 12233, Res Triangle Park, NC 27709, USA. MAPS: A microarray project system for gene expression experiment information and data validation. *Bioinformatics* **17:** (6) 564.

Choo KB, Chen HH, Cheng WTK, Chang HS, Wang M. 2001. Vet Gen Hosp, Dept Med Res & Educ, Recombinant DNA Lab, Taipei 11217, Taiwan. *In silico* mining of EST databases for novel pre-implantation embryo-specific zinc finger protein genes. *Mol Reprod Dev* **59:** (3) 249.

Claverie JM. 2001. CNRS Aventis, UMR 1889, 31 Chem Joseph-Aiguier, FR-13402 Marseille 20, France. Bioinformatics of transcriptome analysis in cancer biology (French, English Abstract). *Bull Cancer* **88:** (3) 269.

Copyright © 2001 John Wiley & Sons, Ltd. 

Cooper CA, Gasteiger E, Packer NH. 2001. Proteome Syst Ltd, Locked Bag 2073, Sydney, NSW 1670, Australia. GlycoMod: A software tool for determining glycosylation compositions from mass spectrometric data. *Proteomics* **1:** (2) 340.
Deane CM, Kaas Q, Blundell TL. 2001. Univ Cambridge, Dept Biochem, Tennis Court Rd, Cambridge CB2 1GA, England. SCORE: Predicting the core of protein models. *Bioinformatics* **17:** (6) 541.
Dysvik B, Jonassen I*. 2001. *Univ Bergen, Dept Informatics, NIB, NO-5020 Bergen, Norway. J-Express: Exploring gene expression data using Java. *Bioinformatics* **17:** (4) 369.
Eyal E, Najmanovich R, Sobolev V, Edelman M. 2001. Weizmann Inst Sci, Dept Plant Sci, IL-76100 Rehovot, Israel. MutaProt: A web interface for structural analysis of point mutations. *Bioinformatics* **17:** (4) 381.
Gaschen B, Kuiken C, Korber B, Foley B. 2001. Los Alamos Natl Lab, HIV Database & Anal Grp, Mail Stop K710, Los Alamos, NM 87545, USA. Retrieval and on-the-fly alignment of sequence fragments from the HIV database. *Bioinformatics* **17:** (5) 415.
Geier B, Kastenmuller G, Fellenberg M, Mewes HW, Morgenstern B*. 2001. *GSF Forschungszentrum Umwelt & Gesundheit, MIPS Inst Bioinformat, GmbH Ingoldstadter Landstr 1, DE-85764 Neuherberg, Germany. The HIB database of annotated UniGene clusters. *Bioinformatics* **17:** (6) 571.
Hill DP, Davis AP, Richardson JE, Corradi JP, Ringwald M, Eppig JT, Blake JA. 2001. Jackson Lab, 600 Main St, Bar Harbor, Me 04609, USA. Strategies for biological annotation of mammalian systems: Implementing gene ontologies in mouse genome informatics. *Genomics* **74:** (1) 121.
Hubbard MJ, Faught MJ, Carlisle BH, Stockwell PA. 2001. Univ Otago, Dept Biochem, 676 Cumberland St, POB 56, Dunedin, New Zealand. ToothPrint, a proteomic database for dental tissues. *Proteomics* **1:** (1) 132.
Jenssen TK, Laegreid A, Komorowski J, Hovig E*. 2001. *Norwegian Radium Hosp, Inst Canc Res, Dept Tumor Biol, Oslo, Norway. A literature network of human genes for high-throughput analysis of gene expression. *Nat Genet* **28:** (1) 21.
Jung E, Veuthey AL, Gasteiger E, Bairoch A*. 2001. *Ctr Med Univ, Inst Suisse Bioinformatics, 1 Michel Servet, CH-1211 Geneva 4, Switzerland. Annotation of glycoproteins in the SWISS-PROT database. *Proteomics* **1:** (2) 262.
Karp PD, Paley S, Zhu JC. 2001. SRI Int, Bioinformatics Research Grp, 333 Ravenswood Ave, Menlo Park, Ca 94025, USA. Database verification studies of SWISS-PROT and GenBank. *Bioinformatics* **17:** (6) 526.
Leung SW, Mellish C, Robertson D. 2001. Univ Edinburgh, Div Informat, 80 Sth Bridge, Edinburgh EH1 1HN, Scotland. Basic Gene Grammars and DNA-ChartParser for language processing of *Escherichia coli* promoter DNA sequences. *Bioinformatics* **17:** (3) 226.
Li WZ, Jaroszewski L, Godzik A*. 2001. *San Diego Supercomp Ctr, La Jolla, Ca 92093, USA. Clustering of highly homologous sequences to reduce the size of large protein databases. *Bioinformatics* **17:** (3) 282.
Loytynoja A, Milinkovitch MC*. 2001. *Free Univ Brussels, Inst Mol Biol & Med, rue Jeener & Brachet 12, BE-6041 Gosselies, Belgium. SOAP, cleaning multiple alignments from unstable blocks. *Bioinformatics* **17:** (6) 573.
Lukashin AV, Fuchs R. 2001. Biogen Inc, 14 Cambridge Ctr, Cambridge, Ma 02142, USA. Analysis of temporal gene expression profiles: Clustering by simulated annealing and determining the optimal number of clusters. *Bioinformatics* **17:** (5) 405.
Marcotte EM, Xenarios I, Eisenberg D. 2001. UCLA, DOE, Lab Struct Biol & Mol Med, Inst Mol Biol, POB 951570, Los Angeles, Ca 90095, USA. Mining literature for protein-protein interactions. *Bioinformatics* **17:** (4) 359.
Masys DR, Welsh JB, Fink JL, Gribskov M, Klacansky I, Corbeil J. 2001. Univ Calif San Diego, Sch Med, Ctr Canc, Dept Med, 9500 Gilman Dr, La Jolla, Ca 92093, USA. Use of keyword hierarchies to interpret gene expression patterns. *Bioinformatics* **17:** (4) 319.
Notredame C. 2001. CNRS UMR 1889, Informat Genet & Struct 31, Ch Joseph Aiguier, FR-13402 Marseille, France. Mocca: Semi automatic method for domain hunting. *Bioinformatics* **17:** (4) 373.
Pauws E, Van Kampen AHC, Van de Graaf SAR, De Vijlder JJM, Ris-Stalpers C. 2001. Univ Amsterdam, Acad Med Ctr, Lab Pediat Endocrinol, POB 22700, NL-1100 DE Amsterdam, The Netherlands. Heterogeneity in polyadenylation cleavage sites in mammalian mRNA sequences: Implications for SAGE analysis. *Nucleic Acids Res* **29:** (8) 1690.
Qi D, Cuticchia AJ*. 2001. *Hosp Sick Children, Bioinformatics Supercomput Ctr, 555 Univ Ave, Toronto, Ontario, Canada M5G 1X8. Compositional symmetries in complete genomes. *Bioinformatics* **17:** (6) 557.
Qin L, Prins P, Jones JT, Popeijus H, Smant G, Bakker J, Helder J. 2001. Univ Wageningen & Res Ctr, Grad Sch Expt Plant Sci, Nematol Lab, Binnenhaven 10, NL-6709 PD Wageningen, The Netherlands. GenEST, a powerful bidirectional link between cDNA sequence data and gene expression profiles generated by cDNA-AFLP. *Nucleic Acids Res* **29:** (7) 1616.
Rogic S, Mackworth AK, Ouellette FBF. 2001. Univ Calif, Dept Comp Sci, Santa Cruz, Ca 95064, USA. Evaluation of gene-finding programs on mammalian sequences. *Genome Res* **11:** (5) 817.
Rognes T. 2001. Univ Oslo, Natl Hosp, Inst Med Microbiol, Dept Mol Biol, NO-0027 Oslo, Norway. ParAlign: A parallel sequence alignment algorithm for rapid and sensitive database searches. *Nucleic Acids Res* **29:** (7) 1647.
Sanchez JC, Chiappe D, Converset V, Hoogland C, Binz PA, Paesano S, Appel RD, Wang S, Sennitt M, Nolan A *et al*. 2001. Hop Cantonal Univ Geneva, Lab Cent Chim Clin, CH-1211 Geneva 14, Switzerland. The mouse SWISS-2D PAGE database: A tool for proteomics study of diabetes and obesity. *Proteomics* **1:** (1) 136.
Soejima H, Kawamoto S, Akai J, Miyoshi O, Arai Y, Morohka T, Matsuo S, Niikawa N, Kimura A, Okubo K, Mukai T. 2001. Saga Med Sch, Dept Biochem, 5-1-1 Nabeshima, Saga 849 9501, Japan. Isolation of novel heart-specific genes using the BodyMap database. *Genomics* **74:** (1) 115.
Spang R, Vingron M. 2001. Duke Univ, Inst Stat & Decision Sci, Box 90251, Durham, NC 27706, USA. Limits of homology detection by pairwise sequence comparison. *Bioinformatics* **17:** (4) 338.
Stoeckert C, Pizarro A, Manduchi E, Gibson M, Brunk B, Crabtree J, Schug J, Shen-Orr S, Overton GC. 2001. Univ Penn, Ctr Bioinformatics, Computat Biol & Informatics Lab, 1313 Blockley Hall, 418 Guardian Dr, Philadelphia, Pa 19104, USA. A relational scheme for both array-based and SAGE gene expression experiments. *Bioinformatics* **17:** (4) 300.
Storm CEV, Sonnhammer ELL. 2001. Karolinska Inst, Ctr Genomics Res, SE-17177 Stockholm, Sweden. NIFAS: Visual analysis of domain evolution in proteins. *Bioinformatics* **17:** (4) 343.
Sujatha S, Balaji S, Srinivasan N*. 2001. *Indian Inst Sci, Mol Biophys Unit, IN-560012 Bangalore, India. PALI: A database for alignments and phylogeny of homologous protein structures. *Bioinformatics* **17:** (4) 375.
Thorn KS, Bogan AA. 2001. Univ Calif San Francisco, Grad Grp Biophys, San Francisco, Ca 94143, USA. ASEdb: A database of alanine mutations and their effects on the free energy of binding in protein interactions. *Bioinformatics* **17:** (3) 284.
Troyanskaya O, Cantor M, Sherlock G, Brown P, Hastie T, Tibshirani R, Botstein D, Altman RB*. 2001. *Stanford Univ, Sch Med, Med Informatics, Stanford, Ca 94305, USA. Missing value estimation methods for DNA microarrays. *Bioinformatics* **17:** (6) 520.
Wall ME, Dyck PA, Brettin TS*. 2001. *Los Alamos Natl Lab, Biosci Div, POB 1663, Los Alamos, NM 87545, USA. SVDMAN: Singular value decomposition analysis of microarray data. *Bioinformatics* **17:** (6) 566.
Ward JM. 2001. Univ Minnesota, Dept Plant Biol, 220 Biosci Ctr, 1445 Gortner Ave, St Paul, Mn, USA. Identification of novel families of membrane proteins from the model plant *Arabidopsis thaliana*. *Bioinformatics* **17:** (6) 560.
Wu TJ, Hsieh YC, Li LA. 2001. Natl Cheng Kung Univ, Dept Stat, Tainan 70101, Taiwan. Statistical measures of DNA sequence dissimilarity under Markov chain models of base composition. *Biometrics* **57:** (2) 441.
Xia XH, Xie Z. 2001. Univ Hong Kong, Dept Ecol & Biodiversity, Hong Kong, Peoples Rep China. AMADA: Analysis of microarray data. *Bioinformatics* **17:** (6) 569.
Yeung KY, Haynor DR, Ruzzo WL. 2001. Univ Washington, Box 352350, Seattle, Wa 98195, USA. Validating clustering for gene expression data. *Bioinformatics* **17:** (4) 309.
Yuan QP, Quackenbush J, Sultana R, Pertea M, Salzberg SL, Buell CR*. 2001. *Inst Genomic Res, 9712 Med Ctr Dr, Rockville, Md 20850, USA. Rice bioinformatics. Analysis of rice sequence data and leveraging the data to other plant species. *Plant Physiol* **125:** (3) 1166

Copyright © 2001 John Wiley & Sons, Ltd. 

